# Zinc Supplementation Inhibits Complement Activation in Age-Related Macular Degeneration

**DOI:** 10.1371/journal.pone.0112682

**Published:** 2014-11-13

**Authors:** Dzenita Smailhodzic, Freekje van Asten, Anna M. Blom, Frida C. Mohlin, Anneke I. den Hollander, Johannes P. H. van de Ven, Ramon A. C. van Huet, Joannes M. M. Groenewoud, Yuan Tian, Tos T. J. M. Berendschot, Yara T. E. Lechanteur, Sascha Fauser, Chris de Bruijn, Mohamed R. Daha, Gert Jan van der Wilt, Carel B. Hoyng, B. Jeroen Klevering

**Affiliations:** 1 Department of Ophthalmology, Radboud university medical center, Nijmegen, the Netherlands; 2 Section of Medical Protein Chemistry, Department of Laboratory Medicine Malmo, Lund University, Malmo, Sweden; 3 Department of Human Genetics, Radboud university medical center, Nijmegen, the Netherlands; 4 Department for Health Evidence, Radboud university medical center, Nijmegen, the Netherlands; 5 University Eye Clinic Maastricht, Maastricht, the Netherlands; 6 Department of Ophthalmology, University of Cologne, Cologne, Germany; 7 Innomedics, Düsseldorf, Germany; 8 Department of Nephrology, Leiden University Medical Center, Leiden, the Netherlands; University of Utah, United States of America

## Abstract

Age-related macular degeneration (AMD) is the leading cause of blindness in the Western world. AMD is a multifactorial disorder but complement-mediated inflammation at the level of the retina plays a pivotal role. Oral zinc supplementation can reduce the progression of AMD but the precise mechanism of this protective effect is as yet unclear. We investigated whether zinc supplementation directly affects the degree of complement activation in AMD and whether there is a relation between serum complement catabolism during zinc administration and the *complement factor H* (*CFH)* gene or the *Age-Related Maculopathy susceptibility 2 (ARMS2)* genotype. In this open-label clinical study, 72 randomly selected AMD patients in various stages of AMD received a daily supplement of 50 mg zinc sulphate and 1 mg cupric sulphate for three months. Serum complement catabolism–defined as the C3d/C3 ratio–was measured at baseline, throughout the three months of supplementation and after discontinuation of zinc administration. Additionally, downstream inhibition of complement catabolism was evaluated by measurement of anaphylatoxin C5a. Furthermore, we investigated the effect of zinc on complement activation *in vitro.* AMD patients with high levels of complement catabolism at baseline exhibited a steeper decline in serum complement activation (p<0.001) during the three month zinc supplementation period compared to patients with low complement levels. There was no significant association of change in complement catabolism and *CFH* and *ARMS2* genotype. *In vitro* zinc sulphate directly inhibits complement catabolism in hemolytic assays and membrane attack complex (MAC) deposition on RPE cells. This study provides evidence that daily administration of 50 mg zinc sulphate can inhibit complement catabolism in AMD patients with increased complement activation. This could explain part of the mechanism by which zinc slows AMD progression.

**Trial Registration:**

The Netherlands National Trial Register NTR2605

## Introduction

Worldwide, age-related macular degeneration (AMD) affects 30–50 million people and is the leading cause of blindness in the Western world [1]–[4]. AMD is a complex, multifactorial disease that manifests clinically as a loss of central vision resulting in an inability to read, recognize faces or discriminate colors. The hallmark lesions of early stage AMD are drusen, which are pathological deposits of extracellular material that form between the retinal pigment epithelium and Bruch membrane [5]. The late stages of AMD can be separated into geographic atrophy and neovascular AMD [5]. In patients with neovascular AMD, choroidal blood vessels invade the central retina and subretinal space causing a rapidly progressive loss of vision [5]. Although the neovascular AMD accounts for 10% of all AMD patients, it is responsible for the majority of AMD-related severe visual impairment [6]–[9]. Despite the beneficial effects of intraocular injections of vascular endothelial growth factor A (VEGF-A) inhibitors [10]–[11], a large percentage of neovascular AMD patients continue to lose vision [12]–[13]. In patients with geographic atrophy, loss of the RPE and photoreceptor cells in the central retina result in a progressive decline of vision at a much slower rate than neovascular AMD [5]. Unfortunately, an effective therapy for treating geographic atrophy has yet to be developed.

Pivotal studies performed during the past decade have changed our understanding of the molecular mechanisms underlying AMD. These findings have led to the exploration of a new therapeutic paradigm for managing AMD, namely the targeting of specific molecular components in the complement pathway [14]–[15]. The complement system is a major component of innate immunity with crucial roles in the first line defense against invading microorganisms, clearance of the apoptotic cells and modulation of the adaptive immune response [16]. There are three pathways of complement activation: the classical, the lectin and the alternative pathway [16]. The most important step of the alternative complement pathway activation is the formation of unstable C3 convertase C3bBb, which cleaves C3 to generate the active fragment C3b. Deposition of C3b on the target surface triggers the effector molecules C3a, C5a and the membrane attack complex (MAC), resulting in inflammation and cell lysis. The discovery that drusen contain proteins of the alternative complement pathway led to the hypothesis that drusen could be involved in local complement-mediated inflammation [17]–[18]. Moreover, the discovery of a strong association between AMD and genetic variants in *CFH* gene, a major inhibitor of the alternative pathway, provided a second line of evidence in support of the inflammation model [19]–[22]. In addition to *CFH*, several other AMD risk variants have been found in genes underlying the alternative pathway [23]–[26]. A third line of evidence supporting complement involvement in AMD came from studies that showed that AMD patients have higher levels of complement activation products in their blood [27]–[30]. However, it is likely to be several years before any of the complement inhibiting drugs will be approved for routine use in clinical practice, assuming they are eventually found to be safe and effective.

In 2001, the data collected from the Age-Related Eye Disease Study (AREDS) revealed that patients who were treated with zinc–either alone or in combination with vitamins–had reduced progression to advanced AMD [31]. Based on these results, AREDS recommends that persons who are older than 55 years of age and who are at risk for developing advanced AMD should consider taking vitamin supplements plus zinc [31]. A report published by the Blue Mountains Eye Study, a population-based study, confirmed the beneficial effect of zinc in AMD patients [32]. The large population-based Rotterdam Study supported the hypothesis of biological interactions between the *CFH* gene Y402H variant and zinc, β-carotene, lutein/zeaxanthin and omega-3 fatty acids and between the *ARMS2* gene A69S variant and zinc and omega-3 fatty acids [33]. As a result of these findings, the Rotterdam Study recommended that clinicians give dietary advice to young individuals who are at risk for AMD [33]. More recently, AREDS2 demonstrated that addition of lutein, zeaxanthin and omega-3 long-chain polyunsaturated fatty acids to the AREDS formulation, did not further reduce risk of progression to advanced AMD [34]. However, exploratory subgroup analyses demonstrated that addition of lutein and zeaxanthin to the AREDS formulation, resulted in a significant reduction of progression to advanced AMD for persons in the lowest quintile of dietary intake, suggesting different treatment effects within subgroups of AMD patients [34]. Despite the widespread use of zinc and antioxidants among AMD patients, the mechanism by which zinc exerts its beneficial effects in AMD patients has not yet been identified. The design of optimal and appropriate therapies require a comprehensive understanding of the factors that drive and delay pathogenesis of AMD. To add to current knowledge we designed the present study to investigate whether zinc affects the activity of the alternative complement pathway in patients with AMD, which might explain how zinc slows AMD progression in subgroups of patients with AMD. Secondly, we correlate the response to zinc supplements to the *CFH* and *ARMS2* genotype status. Lastly, we conducted an in vitro experiment to evaluate whether there is a direct effect of zinc on complement activation.

## Methods

### Study population of the clinical study

**Figure 1:**
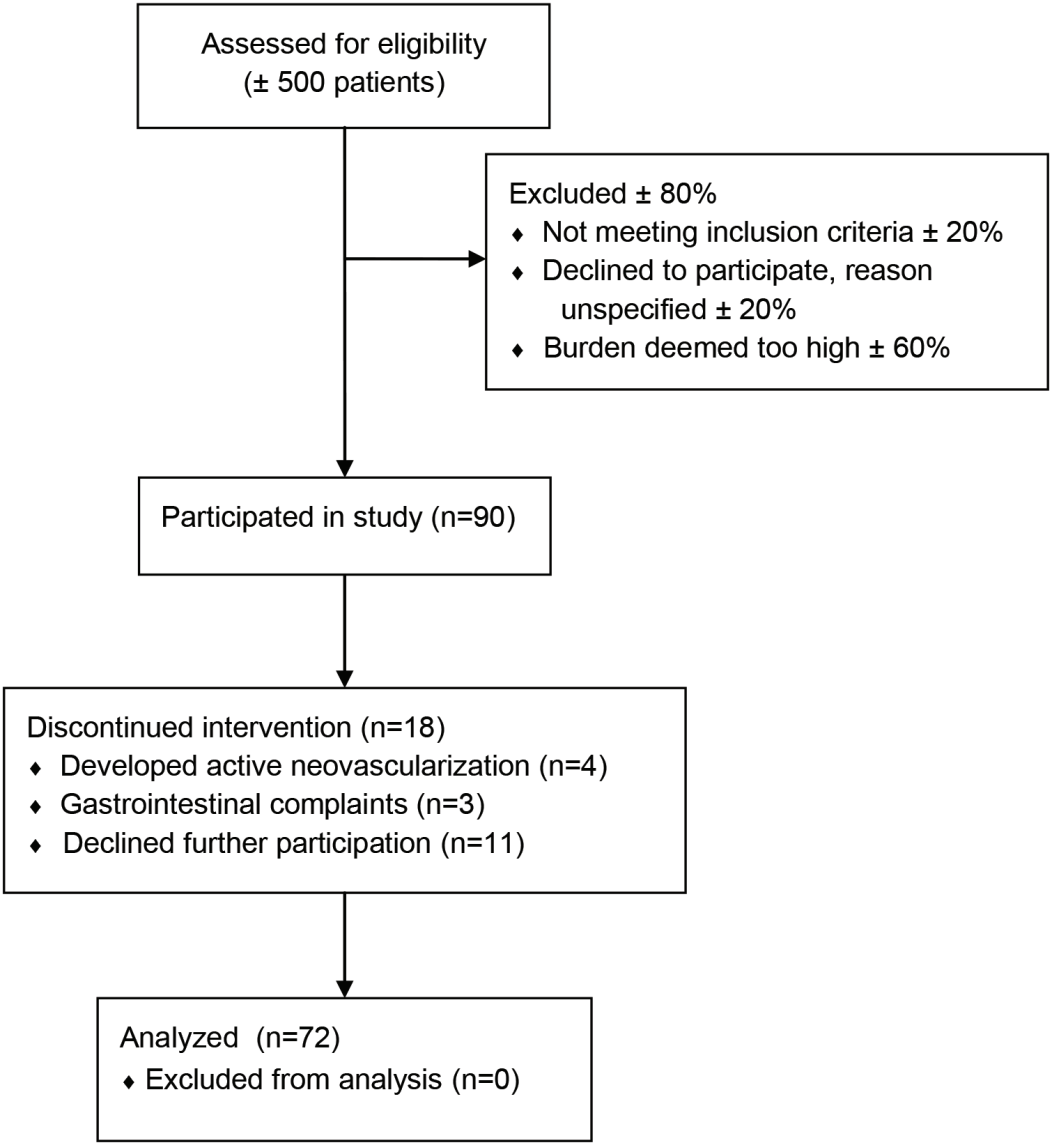
Flow diagram of patient inclusion.

This study was performed in accordance with the Declaration of Helsinki and the Dutch Medical Research Involving Human Subjects Act. Prior to the study, we obtained approval from the local ethics committee (Commissie Mensgebonden Onderzoek regio Arnhem-Nijmegen, April 20^th^ 2010) as well as written informed consent from all participants. This clinical study was registered with The Netherlands National Trial Register (number NTR2605) shortly after recruitment began due to an administrative error. The authors confirm that all ongoing and related trials for this drug/intervention are registered. The protocol for this trial and supporting TREND checklist are available as supporting infromation (Protocol S1 and Checklist S1). The study participants were enrolled in EUGENDA (www.eugenda.org), a multicenter database for the clinical and molecular analysis of AMD, between March 2006 and August 2009. Patients with various stages of AMD were selected at random from the EUGENDA database and were included between June 2010 and February 2011. Follow-up ranged between 14 and 22 months. All data were collected at the department of Ophthalmology of the Radboud university medical center. We excluded individuals who had a core body temperature above 38°C and/or received antibiotics at baseline. In addition, we excluded patients who were receiving intraocular anti-angiogenic treatment, individuals with atypical hemolytic uremic syndrome or membranoproliferative glomerulonephritis type 2 and patients who received local or systemic steroid therapy within the three months prior to the trial. A total of 72 AMD patients were included in this study (Figure 1).

### Study design

To study the effect of zinc on complement activation in patients with AMD, 72 AMD patients received a daily oral supplement containing 50 mg zinc sulphate and 1 mg cupric sulphate in capsule form. The capsules were to be taken at home for a period of three months. These components were donated by Sanmed, Almere, the Netherlands. The 50 mg dose of zinc was lower than in the original AREDS formulation and was chosen to minimize the chance of side-effects. Also, we chose zinc sulphate instead of zinc oxide (as used in the AREDS study), because most over-the-counter supplements contain zinc sulphate and in addition there is evidence that the bioavailability may be higher [35], [36]. The primary endpoint of the study was a change in serum complement catabolism during the three months of zinc supplementation. AMD patients have increased serum levels of C3 and the metabolic byproduct C3d, the most prominent marker of chronic activation of the alternative complement pathway [30]. To correct for individual variations in the level of C3, complement activation was defined as the C3d/C3 ratio as described previously [30]. Anaphylatoxin C5a levels are also elevated in AMD patients and promote choroidal neovascularization [28], [29], [37]. In order to explore downstream inhibitory effects of zinc on complement catabolism, we additionally measured serum C5a levels during the study period. The second objective was to study the association of serum complement catabolism during zinc administration and genotypes of AMD risk variants in *CFH* or *ARMS2.*

During the course of the study, six venous blood samples were collected. One sample was collected prior to zinc supplementation and served as the baseline sample. Three samples were collected at the end of months 1, 2 and 3 of the three-month period of zinc supplementation. We collected a fifth blood sample two months after ending the zinc administration (i.e., at the end of month 5) to check for any reversible effects on complement activation. A final blood sample was collected in months 14–22. From one month prior to zinc supplementation through the end of month 5, the patients were prohibited to take any type of nutritional supplement; from month 5 onwards, the patients were free to take supplements at their own discretion.

To identify clinical manifestations associated with intermittent infections, at every visit, we performed a general physical examination, measured the serum C-reactive protein (CRP) levels and administered a questionnaire that was aimed at identifying clinical manifestations associated with intermittent infections. At every visit, patients were asked whether they had been taking the zinc supplements daily to promote compliance. We also assessed the best-corrected visual acuity using Early-Treatment Diabetic Retinopathy Study (ETDRS) charts at every visit. In addition, we imaged the retinas using high-resolution spectral-domain optical coherence tomography (SD-OCT) to detect active neovascular manifestation of AMD. We performed color fundus photography at baseline to assist in AMD grading based on the 5-grade Clinical Age-Related Maculopathy Staging (CARMS) classification scale [38].

### Complement measurements and genotyping in AMD patients

Serum was prepared by coagulation at room temperature and after centrifugation the samples were stored at −80°C within one hour after collection. C3 and C3d were measured in serum samples as described [39], [40]. All C3 and C3d measurements in this study were performed in a single experiment, except for the final sample in months 14–22. C5a was measured by ELISA at a 1/10 dilution using a commercially available development kit (DuoSet) for human complement component C5a (R&D Systems, Minneapolis, USA). All the samples collected at baseline to month 5 were measured in a single run.

The *CFH* (Y402H; rs1061170) and *ARMS2* (A69S; rs10490924) SNPs were genotyped as described [41]. Serum zinc concentration was measured by atomic absorption spectroscopy with the spectrophotometer 1100 B from Perkin Elmer. CRP levels were measured by Abbott Architect C16000 system. The immunoturbidimetric test for CRP was provided by Abbott Diagnostics (Abbott Diagnostics).

### *In vitro* hemolytic assays and membrane attack complex (MAC) deposition on RPE cells

We designed in vitro experiments to provide evidence of a direct effect of zinc on the complement pathway. Human serum was prepared from blood of several healthy volunteers after written informed consent had been obtained with the specific permit (418/2008) from the ethics committee of Lund University. Commercially available rabbit erythrocytes (Håtunalab, Bro, Sweden) were washed in 2.5 mM veronal buffer pH 7.3, supplemented with 70 mM NaCl, 140 mM glucose, 0.1% porcine gelatin and 7 mM MgCl_2_. Different concentrations (0–64 µM) of zinc sulphate (Merck) were pre-incubated with 2% serum in the same buffer for 1.5 h at 37°C, followed by 1 h incubation at 37°C together with the erythrocytes. The amount of lysed erythrocytes was determined from the amount of released hemoglobin at 405 nm using Cary 50 MPR microplate reader (Varian).

To study the effect of zinc on membrane MAC deposition on human RPE cells subjected to oxidative stress, RPE cells (ARPE-19, ATCC) were cultured in DMEM/F12 media (HyClone), supplemented with 10% FCS (Gibco) and antibiotics (HyClone). After detachment using trypsin, the cells were incubated in medium containing 10 mM H_2_O_2_ for 2 h at 37°C, to mimic oxidative damage and make them amenable to attack from complement [42], [43]. After washing with PBS, the cells were incubated with 5% human serum, together with 0–250 µM zinc sulphate, in the veronal buffer defined above, for 1 h at 37°C. The amount of MAC deposited on the RPE cells was detected using a monoclonal C9 neoepitope antibody (aE11, Hycult), which only recognizes C9 in the C5b-9 complex, followed by a FITC-conjugated secondary antibody and flow cytometric analysis (Partec).

### Statistical analysis

A sample size of 70 was calculated to detect a decrease in serum C3d/C3 of 10% after 3 months, using the complement levels from a previous study to estimate variation [30], with α = 0.05 and a power of 80%.

Change in serum zinc, change in C3d/C3 ratio and change in C5a over the entire study period (0 to 14–22 months) were all modeled separately. Changes in serum zinc concentration were analyzed using a linear mixed-effects model with zinc concentration as the dependent variable. To make optimal use of repeated measures and to allow for correction of baseline differences, changes in C3d/C3 and C5a levels level were analyzed using linear mixed-effects models with C3d/C3 ratio or C5a as the dependent variable. The interaction between time and baseline complement levels was included in a linear mixed-effects model to study any baseline effects. To illustrate the effect of the baseline C3d/C3 ratio we plotted the course of the C3d/C3 ratio for 3 groups with different baseline ratios using the raw data. In a recent study we measured C3d/C3 levels in 150 unaffected control subjects of 65 years and older [30], and we used the mean value (1.5) and standard deviation (0.6) to determine the cut-off points. The cut-off values for the different groups were selected by taking the mean C3d/C3 ratio and one standard deviation above and below the mean of the healthy control group. Our population was not large enough to create groups of individuals with two standard deviations above and below the mean. This resulted in the following three groups: 1. patients with baseline ratio ≥2.1 (n = 16); 2. patients with ratios between 1.5–2.1 (n = 29); and 3. patients with ratio <1.5 (n = 31). Only very few subjects (n = 3) had a baseline ratio more than one standard deviation below the mean, so these individuals were included in group 3. The associations between the complement levels throughout the study and *CFH* and A*RMS2* genotype, age, gender, CRP level and zinc level were also studied using a linear mixed-effects model. In the final models for the change in zinc, C3d/C3 and C5a, only significant predictors were used (p<0.05). For the final serum zinc and C5a models this meant the inclusion of time and baseline values as the independent variables. In the final C3d/C3 model the independent variables were time, baseline C3d/C3 and the interaction between time and baseline C3d/C3.

To further explore the relationship between baseline complement catabolism and other patient characteristics at baseline, we assessed the associations of age and baseline visual acuity with baseline C3d/C3 ratio and C5a using the Pearson correlation and the Spearman’s rank correlation coefficient. The difference in baseline complement catabolism between different genotypes was assessed using one-way ANOVA.

Because patients often display different stages of AMD in each eye, we created five groups for both eyes. These groups were based on the CARMS classification as follows: (CARMS grade 2∶2), small drusen and/or RPE changes in both eyes; (CARMS grade 3∶3), large drusen and/or drusenoid RPE detachment in both eyes; (CARMS grade 2:4–5), small drusen and/or RPE changes in one eye and geographic atrophy or choroidal neovascularisation in the other eye; (CARMS grade 3∶4–5), large drusen and/or drusenoid RPE detachment in one eye and geographic atrophy or choroidal neovascularisation in the other eye; and (CARMS grade 4–5∶4–5), geographic atrophy or choroidal neovascularisation in both eyes. The association between baseline systemic complement catabolism and CARMS classification was tested using one-way ANOVA with a post-hoc Bonferroni correction. The correlation between baseline visual acuity and CARMS was tested using the Spearman’s rank correlation coefficient.

Visual acuity changes during the course of the study were assessed by generalized estimated equations (GEE). The GEE model estimated the probability of low vision (LogMAR <0.5) versus high vision (LogMAR >0.5), with time and baseline C3d/C3 ratio as predictors. Data for the hemolytic assay and the RPE cell assay were analyzed using one-way ANOVA with Dunnett’s multiple comparison test.

Reported p-values are two-sided, and differences were considered to be statistically significant if lower than 0.05. All statistical analyses were performed using SPSS, version 18.0.

## Results

**Figure 2:**
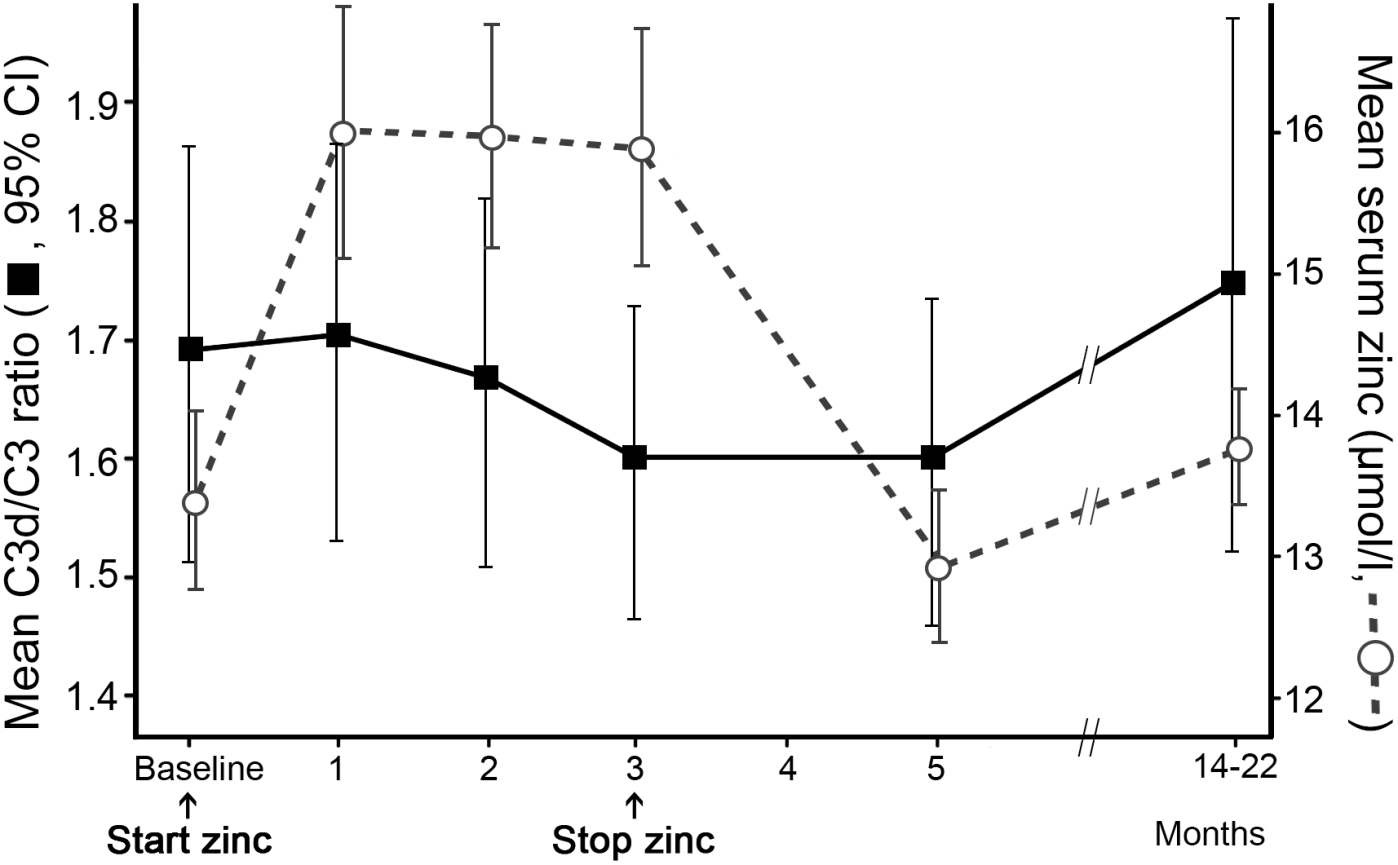
Serum zinc concentration and C3d/C3 ratio throughout the study period. During the three months daily zinc supplementation, serum zinc concentration increased significantly (p<0.001). After zinc supplementation was discontinued, the serum zinc levels returned to baseline levels within 2 months. The C3d/C3 ratio showed non-significant decline during zinc supplementation (p = 0.149).

To evaluate the effect of receiving zinc supplements on systemic complement catabolism, AMD patients received oral zinc sulphate. The baseline characteristics of the study population are presented in Table 1. Serum zinc concentration increased significantly during the supplementation period (p<0.001) and returned to baseline levels two months after the zinc supplements were discontinued (Figure 2). The mean complement activation level, defined as the C3d/C3 ratio, in the 72 patients showed tendency to decline (albeit not significantly; p = 0.149) during the three months of zinc supplementation (Figure 2). From month five onwards, 36 patients indicated they had been using over-the-counter zinc supplements, but generally in lower dosages than used in this study.

**Table 1:** The baseline characteristics of the study population.

Baseline characteristics	AMD, n = 72
Mean age – years ± SD	73.9±8.3
Sex, male – No. (%)	29 (40.3)
Visual acuity OD – median (1^st^–3^rd^ quartile)	20/83 (20/400–20/25)
Visual acuity OS – median (1^st^–3^rd^ quartile)	20/55 (20/333–20/26)
Mean C3d/C3 ratio ± SD	1.65±0.69
Mean zinc level – µmol/l ± SD	13.33±2.83
**CFH (Y402H; rs1061170) genotypes, No. (%)**	
CFH TT genotype (wildtype)	1 (1.4)
CFH CT genotype	36 (50.0)
CFH CC genotype	34 (47.2)
**ARMS2 (A69S; rs10490924) genotypes, No. (%)**	
ARMS2 GG genotype (wildtype)	19 (26.4)
ARMS2 TG genotype	30 (41.7)
ARMS2 TT genotype	22 (30.6)
**Serum C-reactive protein (CRP), No. (%)**	
<5 – mg/l	52 (72.2)
5–15 – mg/l	18 (25.0)
16–45 – mg/l	2 (2.8)

SD = Standard deviation, visual acuity in Snellen.

### Exploration of effect of zinc supplementation on complement catabolism

**Figure 3:**
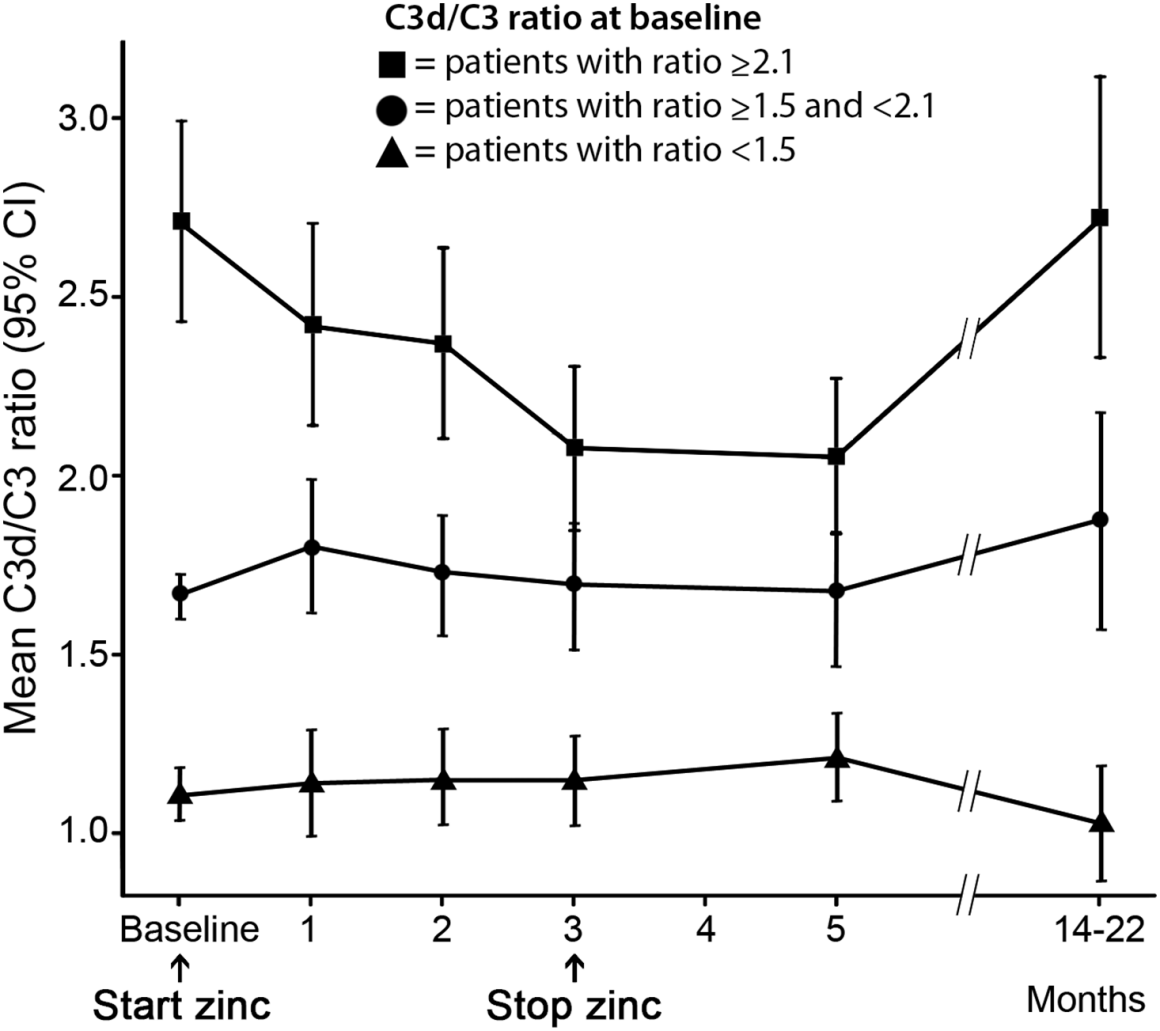
The effect of zinc supplementation on patients with different level of complement catabolism at baseline. The patients with high serum complement catabolism had a steeper decline in C3d/C3 ratio during the administration of zinc sulphate (p<0.001).

**Figure 4:**
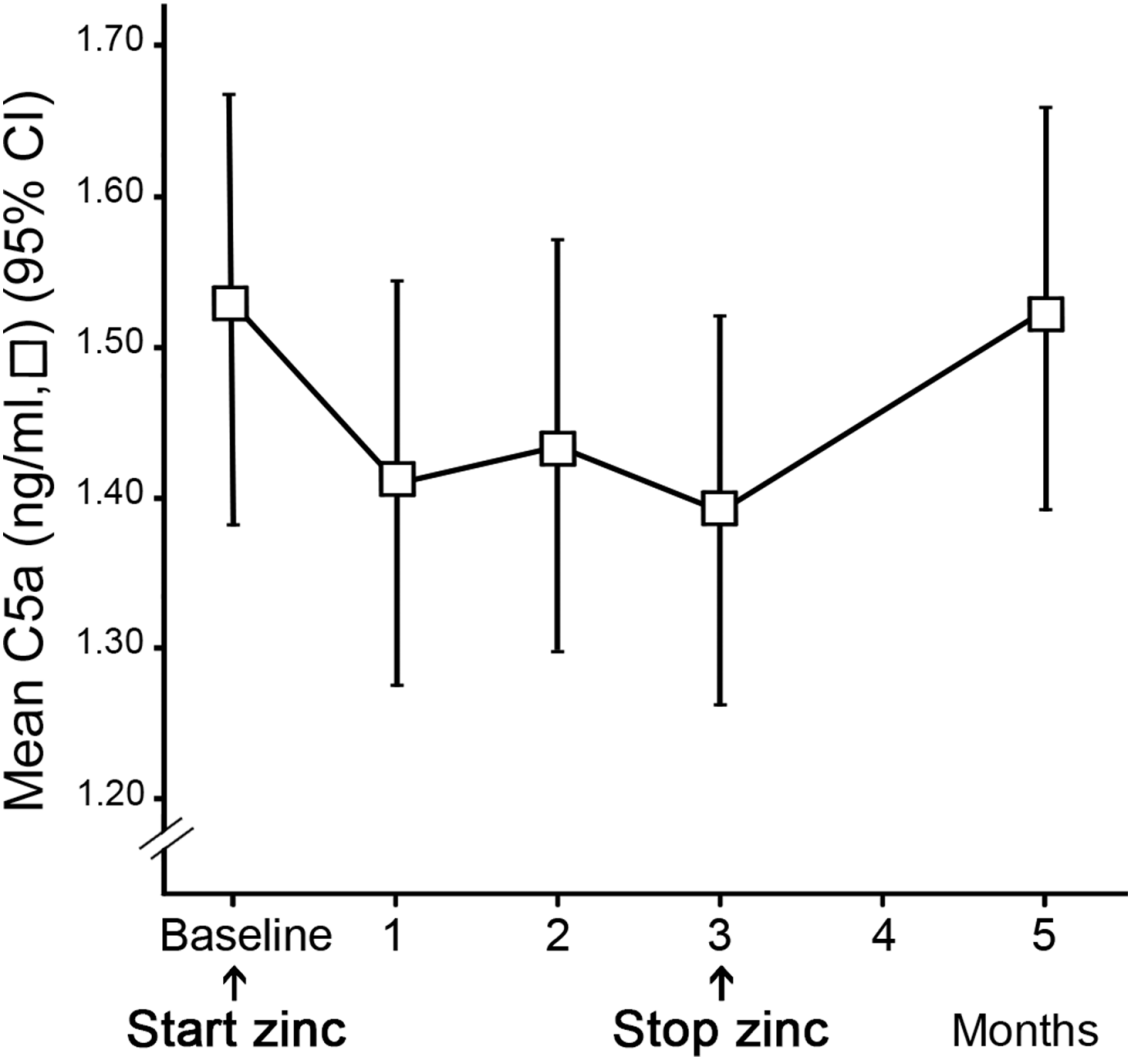
C5a concentration throughout the study period. The C5a levels decreased significantly during the three months of zinc supplementation and returned to baseline level within 2 months after the cessation of zinc supplementation.

We conducted further exploratory analyses whether zinc supplementation may have different effects within patients with different levels of baseline complement catabolism defined as C3d/C3 ratio. In this analysis, we observed a strong interaction between baseline C3d/C3 ratio and change in C3d/C3 ratio during zinc supplementation (p<0.001). The AMD patients with relatively high baseline levels of serum complement catabolism exhibited a more pronounced decline in their C3d/C3 ratio during the administration of zinc sulphate, compared to those AMD patients with lower baseline levels. After the zinc supplementation period, the decline in C3d/C3 ratio remained at this lower level for the following two months. Measurements performed at least nine months later (in months 14–22) showed that complement activation had returned to baseline levels. The AMD patients who already had a relatively low C3d/C3 ratio at baseline showed no decline in C3d/C3 ratio throughout the treatment period. Figure 3 illustrates the course of serum C3d/C3 ratio over time in three groups with different baseline C3d/C3 ratios. The statistical model was not based on these cut-off points. There was no significant association between C3d/C3 ratio and age or gender throughout the course of the study. C5a levels decreased significantly over the three-month supplementation period (p = 0.019) (Figure 4). We observed a similar baseline effect for the course of C5a levels, however, the interaction between baseline and time was not significant and therefore not included in the final model (p = 0.065).

### Association between the stage of AMD and serum complement catabolism

We further analyzed the clinical characteristics of AMD patients with a relatively high baseline complement catabolism. Higher baseline C3d/C3 ratio was significantly associated with younger age (r = −0.33, p = 0.005) and better visual acuity (OD: r = 0.25, p = 0.031 and OS: r = 0.36, p = 0.002). Also, baseline C3d/C3 ratio was associated with the CARMS classification based on both eyes (p = 0.010). Post hoc analysis revealed that patients with large drusen and/or drusenoid RPE detachment in one eye and geographic atrophy or choroidal neovascularization in the other eye (3∶4–5) had higher baseline complement catabolism compared to geographic atrophy or choroidal neovascularization in both eyes (4–5∶4–5) (Table 2). There was no association with baseline C5a and age (r = 0.068, p = 0.583), visual acuity (OD: r = −0.110, p = 0.381 and OS: r = −0.023, p = 0.855) or the CARMS classification based on both eyes (p = 0.947). C3d/C3 ratio and C5a levels were measured in separate experiments and were not correlated (r = 0.086, p = 0.490). As expected, baseline visual acuity for each eye was strongly associated with the CARMS classification per eye (OD: r = −0.69, p<0.001 and OS: r = −0.65, p<0.001) (Table S1).

**Table 2:** Association between the stage of AMD and serum complement catabolism.

Clinical Age-Related Maculopathy Staging (CARMS)for both eyes	Mean C3d/C3ratio (SE)	No. (%)	p*
Grade 2 in both eyes (2:2)	1.64 (0.30)	4 (5.6)	1.000
Grade 2 in the first eye and grade 4 or 5 in thesecond eye (2:4–5)	1.69 (0.31)	9 (12.7)	1.000
Grade 3 in both eyes (3:3)	1.86 (0.17)	10 (14.1)	0.263
Grade 3 in the first and stages 4 or 5 in the secondeye (3:4–5)	2.01 (0.20)	19 (26.8)	0.006
Grades 4 or 5 in both eyes (4–5:4–5)	1.32 (0.06)	29 (40.8)	Ref.

Compared to the patients with intermediate AMD in one eye and late AMD in the other eye (CARMS stage 3∶4–5), the patients who had late AMD in both eyes (CARMS 4–5∶4–5) had significantly lower C3d/C3 levels (p = 0.006).

*p-value from one-way ANOVA with post hoc Bonferroni correction.

### Correlation between the serum complement catabolism and the genotype

The baseline C3d/C3 ratios did not differ significantly between wildtype/heterozygous Y402H *CFH* genotype and the homozygous Y402H genotype (p = 0.934) nor between *ARMS2* genotypes (p = 0.729). There was also no difference in baseline C5a between *CFH* (p = 0.597) and *ARMS2* genotypes (p = 0.412). Change in C3d/C3 ratio or C5a levels were not related to *CFH* or *ARMS2* genotypes.

### Intermittent infections and the C3d/C3 ratio

Serum CRP levels were measured at every visit and were not significantly associated with the C3d/C3 ratio (p = 0.168) nor the C5a levels (p = 0.942). Questionnaires demonstrated that antibiotics were prescribed in 10 patients during the study period. Use of antibiotics was not related to increased CRP levels, increased body temperature or elevated C3d/C3 ratio (data not shown) in these individuals.

### Effect of zinc on complement catabolism *in vitro*

**Figure 5:**
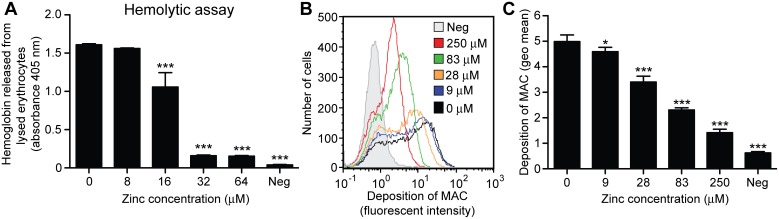
The effect of zinc on the hemolytic activity of human serum and on the membrane attack complex (MAC) deposition on retinal pigment epithelial (RPE) cells. (**A**) Zinc sulphate inhibits the lysis of rabbit erythrocytes in a dose-dependent manner. (**B–C**) the amount of MAC deposited on RPE cells exposed to oxidative stress can be reduced in a dose dependent manner by zinc. *p<0.05 and ***p<0.001.

To demonstrate the *in vitro* effect of zinc on the complement activity of human serum and to better understand the effect observed in vivo in the patients, we performed an alternative pathway hemolytic assay. Results showed that zinc sulphate inhibits the lysis of rabbit erythrocytes in a dose-dependent manner (Figure 5A). Retina is exposed to high levels of oxidative stress from light exposure and metabolic processes [44]. We tested *in vitro* whether zinc could also protect the RPE from a oxidative stress related damage from the complement system. The test results show that the amount of MAC deposited on RPE cells exposed to oxidative stress can be reduced in a dose dependent manner by zinc sulphate (Figure 5B–C). In the negative controls, zinc and serum were omitted.

## Discussion

In the past decade, it has become increasingly clear that complement-mediated inflammation plays a fundamental role in the etiology of AMD [18], [45]. All current therapies for treating neovascular AMD are designed to reduce the ongoing VEGF stimulus–and hence inhibit the growth of new vessels–but do not address the underlying pathology. Moreover, no effective therapy has been developed for treating early AMD or geographic atrophy. The discovery of complement as a major contributing factor to AMD pathogenesis has sparked considerable interest in this system as a potential therapeutic target, and various complement inhibitors are currently being tested in clinical trials [14], [15].

Our findings suggest that increased complement catabolism, defined as the C3d/C3 ratio, in AMD patients can be reduced by the daily oral administration of 50 mg zinc sulphate. However, the effect of complement inhibition seemed to be limited to patients with a high baseline level of complement catabolism. The C3d/C3 ratio returned to its baseline value after the supplementation period, indicating a reversible effect of zinc supplementation on complement activation. Continuous zinc supplementation may therefore be necessary to inhibit complement activity over longer periods of time. Approximately 50% of patients indicated they had been using zinc supplements during the period before the last measurement. However, the zinc dosage and possibly compliance in these patients was apparently too low to exert a clear effect on complement levels.

We then linked the degree of serum complement activation to the clinical stages of AMD and found that the level of serum complement activation is correlated with patients having large drusen and/or drusenoid RPE detachment. It has been demonstrated that 42% of patients with drusenoid RPE detachment progress to end-stage AMD and develop profound and irreversible visual loss within five years [46]. The AREDS1 study showed that this group in particular benefits from zinc plus antioxidant supplementation. Our results suggest that this may be related to increased activation of the alternative complement pathway in this group, which would support the notion that patients with large drusen and/or drusenoid RPE detachment should receive supplements. Although correlation coefficients were modest, higher complement levels at baseline were mostly observed in younger patients with better visual acuity, corresponding with less advanced disease. This could indicate that the use of supplements should not be postponed until more advanced stages of the disease.

C5a showed a significant decrease during zinc supplementation, indicating that zinc inhibition of the complement pathway can also be detected further downstream. We observed a similar pattern of increased inhibitory effect in patients with higher baseline C5a levels, but this was not as profound as for the C3d/C3 ratio. This could be explained by the more unstable nature of C5a as compared to the C3d/C3 ratio which corrects for intrapersonal fluctuations. C5a returned to its baseline value within 2 months after the supplementation period also suggesting a reversible effect of zinc on C5a.

The exact role of genotype in the response to zinc and antioxidant supplements remains unclear. The Rotterdam study showed that high dietary zinc intake reduces the risk of AMD associated with the *CFH* Y402H variant, suggesting a relationship between zinc and this genotype [33]. A recent subgroup analysis, utilizing data from the AREDS study, showed that the response to zinc and antioxidants may be influenced by *CFH* and *ARMS2* genotype. Their results suggested that patients carrying the *CFH* Y402H risk allele have no benefit from zinc supplementation on the 10-year disease progression [47]. In previous studies by AREDS study researchers on the interaction between genotype and treatment response, they found that the AREDS supplements may be less effective in reducing progression in carriers of the *CFH* risk allele [48]. But in a later publication they could not corroborate the interaction and could not find any relation with response to zinc or antioxidants and genotype [49]. Notably, a biochemical study of zinc and factor H showed that the interaction between zinc and the factor H protein was not influenced by the *CFH* Y402H variant [50]. In our study, genotype did not have an effect on baseline or change of complement activation levels. Given the small number of study participants our study probably lacks the power to detect a possible interaction between *CFH* or *ARMS2* genotype and zinc supplementation.

Changes in C3 activation over time can also be caused by various factors related to immune defense in case of infection [51]. Since serum CRP levels were not significantly associated with C3d/C3 ratio, it is unlikely that the observed change in complement catabolism can be ascribed to an intermittent infection. Data obtained from a general physical examination and a questionnaire aimed at identifying clinical manifestations of intermittent infections also did not point to an infectious cause for the change in complement levels in these AMD patients. Finally, it is unlikely that the study results were influenced by the statistical phenomenon of ‘regression to the mean’ because the C3d/C3 ratio returned to baseline levels for after discontinuation of zinc administration.

In further support of our hypothesis that zinc administration affects complement catabolism, we demonstrated *in vitro* that zinc sulphate directly inhibits complement activation in human serum in a dose-dependent manner. In addition, we demonstrated that during oxidative challenge the presence of zinc sulphate diminishes MAC deposition on RPE cells, thereby preventing complement-mediated cytolysis and apoptosis. This implies that zinc not only has the ability to reduce systemic activation of the alternative complement pathway, but may also diminish complement activation locally on RPE cells. Important to note is that zinc concentrations were in physiological levels [44], [52], and therefore have biomedical significance. A previous biochemical study showed that oligomerization of the CFH protein occurs in the presence of zinc, theoretically leading to increased complement activation [53]. A more recent biochemical study from researchers of the same study group demonstrated that factor H-C3b complexes are precipitated by zinc which would inhibit complement activation [54]. Thus we cannot pinpoint the exact molecular mechanism behind our observations, however, we can conclude that zinc inhibits systemic complement activation and local MAC deposition preventing RPE cell damage.

This study has some limitations that should be addressed. A relatively small number of subjects were included, and zinc was administered for a relatively brief period of time. Patient compliance was monitored through questionnaires, which could potentially underestimate zinc intake. However, a steep increase in serum zinc following the initiation of treatment indicated that supplementation had been successful. Because of the slow natural progression of AMD, this study was never designed to measure a direct protective effect of zinc on visual acuity. Larger patient cohorts and a longer period of zinc supplementation should also be studied to corroborate and extend our findings.

In summary, in our study increased levels of serum complement catabolism correlates with the stage of AMD. Our study demonstrate that increased levels of complement catabolism can be normalized by the daily oral administration of 50 mg zinc sulphate. Findings from the present study might explain how zinc slows AMD progression in subgroups of patients with AMD.

## Supporting Information

Table S1 **Visual acuity per CARMS classification grade.** Baseline median visual acuity in Snellen per Clinical Age-Related Maculopathy Staging (CARMS) classification grade for the separate eyes. Visual acuity decreases as CARMS classification increases.
(DOCX)

Checklist S1 **TREND Checklist.**
(DOCX)

Protocol S1 **Clinical trial protocol.**
(PDF)

## References

[1] Kawasaki R, Yasuda M, Song SJ, Chen SJ, Jonas JB, et al (2010) The prevalence of age-related macular degeneration in Asians: a systematic review and meta-analysis. Ophthalmology 117: 921–927. 20110127 10.1016/j.ophtha.2009.10.007

[2] Krishnan T, Ravindran RD, Murthy GV, Vashist P, Fitzpatrick KE, et al (2010) Prevalence of early and late age-related macular degeneration in India: the INDEYE study. Invest Ophthalmol Vis Sci 51: 701–707. 19696177 10.1167/iovs.09-4114 PMC2868454

[3] Rein DB, Wittenborn JS, Zhang X, Honeycutt AA, Lesesne SB, et al (2009) Forecasting age-related macular degeneration through the year 2050: the potential impact of new treatments. Arch Ophthalmol 127: 533–540. 19365036 10.1001/archophthalmol.2009.58

[4] Smith W, Assink J, Klein R, Mitchell P, Klaver CC, et al (2001) Risk factors for age-related macular degeneration: Pooled findings from three continents. Ophthalmology 108: 697–704. 11297486 10.1016/s0161-6420(00)00580-7

[5] de Jong PT (2006) Age-related macular degeneration. N Engl J Med 355: 1474–1485. 17021323 10.1056/NEJMra062326

[6] Friedman DS, O’Colmain BJ, Munoz B, Tomany SC, McCarty C, et al (2004) Prevalence of age-related macular degeneration in the United States. Arch Ophthalmol 122: 564–572. 15078675 10.1001/archopht.122.4.564

[7] Klaver CC, Wolfs RC, Vingerling JR, Hofman A, de Jong PT (1998) Age-specific prevalence and causes of blindness and visual impairment in an older population: the Rotterdam Study. Arch Ophthalmol 116: 653–658. 9596502 10.1001/archopht.116.5.653

[8] Klein R, Klein BE, Tomany SC, Meuer SM, Huang GH (2002) Ten-year incidence and progression of age-related maculopathy: The Beaver Dam eye study. Ophthalmology 109: 1767–1779. 12359593 10.1016/s0161-6420(02)01146-6

[9] Mitchell P, Smith W, Attebo K, Wang JJ (1995) Prevalence of age-related maculopathy in Australia. The Blue Mountains Eye Study. Ophthalmology 102: 1450–1460. 9097791 10.1016/s0161-6420(95)30846-9

[10] Martin DF, Maguire MG, Ying GS, Grunwald JE, Fine SL, et al (2011) Ranibizumab and bevacizumab for neovascular age-related macular degeneration. N Engl J Med 364: 1897–1908. 21526923 10.1056/NEJMoa1102673 PMC3157322

[11] Rosenfeld PJ, Brown DM, Heier JS, Boyer DS, Kaiser PK, et al (2006) Ranibizumab for neovascular age-related macular degeneration. N Engl J Med 355: 1419–1431. 17021318 10.1056/NEJMoa054481

[12] Ip MS, Scott IU, Brown GC, Brown MM, Ho AC, et al (2008) Anti-vascular endothelial growth factor pharmacotherapy for age-related macular degeneration: a report by the American Academy of Ophthalmology. Ophthalmology 115: 1837–1846. 18929163 10.1016/j.ophtha.2008.08.012

[13] Smailhodzic D, Muether PS, Chen J, Kwestro A, Zhang AY, et al (2012) Cumulative effect of risk alleles in CFH, ARMS2, and VEGFA on the response to ranibizumab treatment in age-related macular degeneration. Ophthalmology 119: 2304–2311. 22840423 10.1016/j.ophtha.2012.05.040

[14] Troutbeck R, Al-Qureshi S, Guymer RH (2012) Therapeutic targeting of the complement system in age-related macular degeneration: a review. Clin Experiment Ophthalmol 40: 18–26. 22304025 10.1111/j.1442-9071.2011.02581.x

[15] Yehoshua Z, de Amorim Garcia Filho CA, Nunes RP, Gregori G, Penha FM, et al (2014) Systemic complement inhibition with eculizumab for geographic atrophy in age-related macular degeneration: the COMPLETE study. Ophthalmology 121: 693–701. 24289920 10.1016/j.ophtha.2013.09.044 PMC4015213

[16] Walport MJ (2001) Complement. First of two parts. N Engl J Med 344: 1058–1066. 11287977 10.1056/NEJM200104053441406

[17] Anderson DH, Mullins RF, Hageman GS, Johnson LV (2002) A role for local inflammation in the formation of drusen in the aging eye. Am J Ophthalmol 134: 411–431. 12208254 10.1016/s0002-9394(02)01624-0

[18] Anderson DH, Radeke MJ, Gallo NB, Chapin EA, Johnson PT, et al (2010) The pivotal role of the complement system in aging and age-related macular degeneration: hypothesis re-visited. Prog Retin Eye Res 29: 95–112. 19961953 10.1016/j.preteyeres.2009.11.003 PMC3641842

[19] Edwards AO, Ritter R 3rd, Abel KJ, Manning A, Panhuysen C, et al (2005) Complement factor H polymorphism and age-related macular degeneration. Science 308: 421–424. 15761121 10.1126/science.1110189

[20] Hageman GS, Anderson DH, Johnson LV, Hancox LS, Taiber AJ, et al (2005) A common haplotype in the complement regulatory gene factor H (HF1/CFH) predisposes individuals to age-related macular degeneration. Proc Natl Acad Sci U S A 102: 7227–7232. 15870199 10.1073/pnas.0501536102 PMC1088171

[21] Haines JL, Hauser MA, Schmidt S, Scott WK, Olson LM, et al (2005) Complement factor H variant increases the risk of age-related macular degeneration. Science 308: 419–421. 15761120 10.1126/science.1110359

[22] Klein RJ, Zeiss C, Chew EY, Tsai JY, Sackler RS, et al (2005) Complement factor H polymorphism in age-related macular degeneration. Science 308: 385–389. 15761122 10.1126/science.1109557 PMC1512523

[23] Fagerness JA, Maller JB, Neale BM, Reynolds RC, Daly MJ, et al (2009) Variation near complement factor I is associated with risk of advanced AMD. Eur J Hum Genet 17: 100–104. 18685559 10.1038/ejhg.2008.140 PMC2985963

[24] Gold B, Merriam JE, Zernant J, Hancox LS, Taiber AJ, et al (2006) Variation in factor B (BF) and complement component 2 (C2) genes is associated with age-related macular degeneration. Nat Genet 38: 458–462. 16518403 10.1038/ng1750 PMC2921703

[25] Maller JB, Fagerness JA, Reynolds RC, Neale BM, Daly MJ, et al (2007) Variation in complement factor 3 is associated with risk of age-related macular degeneration. Nat Genet 39: 1200–1201. 17767156 10.1038/ng2131

[26] Yates JR, Sepp T, Matharu BK, Khan JC, Thurlby DA, et al (2007) Complement C3 variant and the risk of age-related macular degeneration. N Engl J Med 357: 553–561. 17634448 10.1056/NEJMoa072618

[27] Hecker LA, Edwards AO, Ryu E, Tosakulwong N, Baratz KH, et al (2010) Genetic control of the alternative pathway of complement in humans and age-related macular degeneration. Hum Mol Genet 19: 209–215. 19825847 10.1093/hmg/ddp472 PMC2792151

[28] Reynolds R, Hartnett ME, Atkinson JP, Giclas PC, Rosner B, et al (2009) Plasma complement components and activation fragments: associations with age-related macular degeneration genotypes and phenotypes. Invest Ophthalmol Vis Sci 50: 5818–5827. 19661236 10.1167/iovs.09-3928 PMC2826794

[29] Scholl HP, Charbel Issa P, Walier M, Janzer S, Pollok-Kopp B, et al (2008) Systemic complement activation in age-related macular degeneration. PLoS One 3: e2593. 18596911 10.1371/journal.pone.0002593 PMC2440421

[30] Smailhodzic D, Klaver CC, Klevering BJ, Boon CJ, Groenewoud JM, et al (2012) Risk alleles in CFH and ARMS2 are independently associated with systemic complement activation in age-related macular degeneration. Ophthalmology 119: 339–346. 22133792 10.1016/j.ophtha.2011.07.056

[31] Age-Related Eye Disease Study Research Group (2001) A randomized, placebo-controlled, clinical trial of high-dose supplementation with vitamins C and E, beta carotene, and zinc for age-related macular degeneration and vision loss: AREDS report no. 8. Arch Ophthalmol 119: 1417–1436. 11594942 10.1001/archopht.119.10.1417 PMC1462955

[32] Tan JS, Wang JJ, Flood V, Rochtchina E, Smith W, et al (2008) Dietary antioxidants and the long-term incidence of age-related macular degeneration: the Blue Mountains Eye Study. Ophthalmology 115: 334–341. 17664009 10.1016/j.ophtha.2007.03.083

[33] Ho L, van Leeuwen R, Witteman JC, van Duijn CM, Uitterlinden AG, et al (2011) Reducing the genetic risk of age-related macular degeneration with dietary antioxidants, zinc, and omega-3 fatty acids: the Rotterdam study. Arch Ophthalmol 129: 758–766. 21670343 10.1001/archophthalmol.2011.141

[34] Age-Related Eye Disease Study 2 Research Group (2013) Lutein + zeaxanthin and omega-3 fatty acids for age-related macular degeneration: the Age-Related Eye Disease Study 2 (AREDS2) randomized clinical trial. JAMA 309: 2005–2015. 23644932 10.1001/jama.2013.4997

[35] Wedekind KJ, Baker DH (1990) Zinc bioavailability in feed-grade sources of zinc. J Anim Sci 68: 684–689. 2318732 10.2527/1990.683684x

[36] Schell TC, Kornegay ET (1996) Zinc concentration in tissues and performance of weanling pigs fed pharmacological levels of zinc from ZnO, Zn-methionine, Zn-lysine, or ZnSO4. J Anim Sci 74: 1584–1593. 8818803 10.2527/1996.7471584x

[37] Nozaki M, Raisler BJ, Sakurai E, Sarma JV, Barnum SR, et al (2006) Drusen complement components C3a and C5a promote choroidal neovascularization. Proc Natl Acad Sci U S A 103: 2328–2333. 16452172 10.1073/pnas.0408835103 PMC1413680

[38] Seddon JM, Sharma S, Adelman RA (2006) Evaluation of the clinical age-related maculopathy staging system. Ophthalmology 113: 260–266. 16458093 10.1016/j.ophtha.2005.11.001

[39] Reddingius RE, Schroder CH, Daha MR, Monnens LA (1993) The serum complement system in children on continuous ambulatory peritoneal dialysis. Perit Dial Int 13: 214–218. 8369352

[40] Siezenga MA, Chandie Shaw PK, van der Geest RN, Mollnes TE, Daha MR, et al (2009) Enhanced complement activation is part of the unfavourable cardiovascular risk profile in South Asians. Clin Exp Immunol 157: 98–103. 19659775 10.1111/j.1365-2249.2009.03959.x PMC2710597

[41] Hawkins JR, Khripin Y, Valdes AM, Weaver TA (2002) Miniaturized sealed-tube allele-specific PCR. Hum Mutat 19: 543–553. 11968087 10.1002/humu.10060

[42] Kim MH, Chung J, Yang JW, Chung SM, Kwag NH, et al (2003) Hydrogen peroxide-induced cell death in a human retinal pigment epithelial cell line, ARPE-19. Korean J Ophthalmol 17: 19–28. 12882504 10.3341/kjo.2003.17.1.19

[43] Bandyopadhyay M, Rohrer B (2012) Matrix metalloproteinase activity creates pro-angiogenic environment in primary human retinal pigment epithelial cells exposed to complement. Invest Ophthalmol Vis Sci 53: 1953–1961. 22408008 10.1167/iovs.11-8638 PMC3995566

[44] Wills NK, Ramanujam VM, Kalariya N, Lewis JR, van Kuijk FJ (2008) Copper and zinc distribution in the human retina: relationship to cadmium accumulation, age, and gender. Exp Eye Res 87: 80–88. 18579132 10.1016/j.exer.2008.04.013

[45] Charbel Issa P, Chong NV, Scholl HP (2011) The significance of the complement system for the pathogenesis of age-related macular degeneration - current evidence and translation into clinical application. Graefes Arch Clin Exp Ophthalmol 249: 163–174. 21127893 10.1007/s00417-010-1568-6 PMC3042099

[46] Cukras C, Agron E, Klein ML, Ferris FL 3rd, Chew EY, et al (2010) Natural history of drusenoid pigment epithelial detachment in age-related macular degeneration: Age-Related Eye Disease Study Report No. 28. Ophthalmology 117: 489–499. 20079925 10.1016/j.ophtha.2009.12.002 PMC2947750

[47] Awh CC, Lane AM, Hawken S, Zanke B, Kim IK (2013) CFH and ARMS2 genetic polymorphisms predict response to antioxidants and zinc in patients with age-related macular degeneration. Ophthalmology 120: 2317–2323. 23972322 10.1016/j.ophtha.2013.07.039

[48] Klein ML, Francis PJ, Rosner B, Reynolds R, Hamon SC, et al (2008) CFH and LOC387715/ARMS2 genotypes and treatment with antioxidants and zinc for age-related macular degeneration. Ophthalmology 115: 1019–1025. 18423869 10.1016/j.ophtha.2008.01.036

[49] Chew EY, Klein ML, Clemons TE, Agron E, Ratnapriya R, et al (2014) No Clinically Significant Association between CFH and ARMS2 Genotypes and Response to Nutritional Supplements: AREDS Report Number 38. Ophthalmology. 10.1016/j.ophtha.2014.05.008 PMC4253656 24974817

[50] Nan R, Farabella I, Schumacher FF, Miller A, Gor J, et al (2011) Zinc binding to the Tyr402 and His402 allotypes of complement factor H: possible implications for age-related macular degeneration. J Mol Biol 408: 714–735. 21396937 10.1016/j.jmb.2011.03.006 PMC3092982

[51] Zipfel PF, Skerka C (2009) Complement regulators and inhibitory proteins. Nat Rev Immunol 9: 729–740. 19730437 10.1038/nri2620

[52] Lowe NM, Medina MW, Stammers AL, Patel S, Souverein OW, et al (2012) The relationship between zinc intake and serum/plasma zinc concentration in adults: a systematic review and dose-response meta-analysis by the EURRECA Network. Br J Nutr 108: 1962–1971. 23244547 10.1017/S0007114512004382

[53] Nan R, Gor J, Lengyel I, Perkins SJ (2008) Uncontrolled zinc- and copper-induced oligomerisation of the human complement regulator factor H and its possible implications for function and disease. J Mol Biol 384: 1341–1352. 18976665 10.1016/j.jmb.2008.10.030

[54] Nan R, Tetchner S, Rodriguez E, Pao PJ, Gor J, et al (2013) Zinc-induced self-association of complement C3b and Factor H: implications for inflammation and age-related macular degeneration. J Biol Chem 288: 19197–19210. 23661701 10.1074/jbc.M113.476143 PMC3696691

