# Supplementary material for: Zinc Supplementation Inhibits Complement Activation in Age-Related Macular Degeneration
Source: PLoS One. 2014 Nov 13;9(11):e112682. doi: 10.1371/journal.pone.0112682 (PMC4231060; doi:10.1371/journal.pone.0112682)
Supplement: Table S1 — Visual acuity per CARMS classification grade. Baseline median visual acuity in Snellen per Clinical Age-Related Maculopathy Staging (CARMS) classification grade for the separate eyes. Visual acuity decreases as CARMS classification increases. (DOCX) [file pone.0112682.s001.docx]

**Table S1. Visual acuity per CARMS classification grade.**

Baseline median visual acuity in Snellen per Clinical Age-Related Maculopathy Staging (CARMS) classification grade for the separate eyes. Visual acuity decreases as CARMS classification increases.

| **Clinical Age-Related Maculopathy Staging**  **(CARMS)** | **Visual acuity, median (1^st^-3^rd^ quartile)** | |
| --- | --- | --- |
|  | **OD** | **OS** |
| Grade 2 | 20/20 (20/25-20/20) | 20/22 (20/40-20/20) |
| Grade 3 | 20/25 (20/40-20/21) | 20/25 (20/50-20/20) |
| Grade 4 | 20/143 (20/200-20/40) | 20/63 (20/200-20/40) |
| Grade 5 | 20/400 (20/1000-20/133) | 20/267 (20/1000-20/80) |
| Visual acuity in Snellen |  |  |
