# Supplementary material for: Zinc Supplementation Inhibits Complement Activation in Age-Related Macular Degeneration
Source: PLoS One. 2014 Nov 13;9(11):e112682. doi: 10.1371/journal.pone.0112682 (PMC4231060; doi:10.1371/journal.pone.0112682)
Supplement: Protocol S1 — Clinical trial protocol. (PDF) [file pone.0112682.s003.pdf]

RESEARCH PROTOCOL

Serum complement levels and the relation between zinc and Age-Related Macular Degeneration

PROTOCOL TITLE: Serum complement levels and the relation between zinc and Age-Related Macular Degeneration

**Protocol ID**	***<include protocol ID given by sponsor or investigator>***
**Short title**	Zink supplementation in AMD
**Version**	1.2
**Date**	16-02-2010
**Coordinating investigator/project leader**	D. Smailhodzic<br>Radboud University Nijmegen Medical Centre<br>P.O. Box 9101, 6500 HB Nijmegen<br>The Netherland,tel: +31 (0)24-3615172
**Principal investigator(s) (in Dutch: hoofdonderzoeker/uitvoerder)**<br>***Multicenter research: per site***	B. J. Klevering<br>Radboud University Nijmegen Medical Centre<br>P.O. Box 9101, 6500 HB Nijmegen<br>The Netherlands, tel: +31 (0)24-3613138
**Sponsor (in Dutch: verrichter/opdrachtgever)**	B. J. Klevering<br>Radboud University Nijmegen Medical Centre<br>P.O. Box 9101, 6500 HB Nijmegen<br>The Netherlands, tel: +31 (0)24-3613138
**Independent physician(s)**	N. Crama<br>Radboud University Nijmegen Medical Centre<br>P.O. Box 9101, 6500 HB Nijmegen<br>The Netherlands, tel: +31 (0)24-3613138
**Laboratory sites**	M. Daha<br>Leids Universitair Medisch Centrum<br>Albinusdreef 2, 2333 ZA Leiden<br>The Netherlands, tel: +31 (0)71-5263964
**Pharmacy**	Sanmed BV<br>Damsluisweg 48, 1332 ED, Almere<br>The Netherlands ,tel. 036-5476040

PROTOCOL SIGNATURE SHEET

Name	Signature	Date
**Sponsor or legal representative:**<br>**For non-commercial research,**<br>**Head of Department:**<br>*J.J.E. Keunen, MD, PhD*		**16-02-2010**
**Coordinating Investigator/Project leader/Principal Investigator:**<br>*D. Smailhodzic, MD*		**16-02-2010**

TABLE OF CONTENTS

1. INTRODUCTION AND RATIONALE 10
2. OBJECTIVES 11
3. STUDY DESIGN 12
STUDY POPULATION 12
4.1 Population (base) 12
4.2 Inclusion criteria 12
4.3 Exclusion criteria 13
4.4 Sample size calculation 13
5. TREATMENT OF SUBJECTS 14
5.1 Investigational product/treatment 14
5.2 Use of co-intervention (if applicable) 14
5.3 Escape medication (if applicable) 15
6. INVESTIGATIONAL MEDICINAL PRODUCT 15
6.1 Name and description of investigational medicinal product 15
6.2 Summary of findings from non-clinical studies 15
6.3 Summary of findings from clinical studies 15
6.4 Summary of known and potential risks and benefits 16
6.5 Description and justification of route of administration and dosage 17
6.6 Dosages, dosage modifications and method of administration 17
6.7 Preparation and labelling of Investigational Medicinal Product 18
6.8 Drug accountability 18
7. METHODS 18
7.1 Study parameters/endpoints 18
7.1.1 Main study parameter/endpoint 18
7.1.2 Secondary study parameters/endpoints (if applicable) 19
7.1.3 Other study parameters (if applicable) 19
7.2 Randomisation, blinding and treatment allocation 19
7.3 Study procedures 19
7.4 Withdrawal of individual subjects 21
7.4.1 Specific criteria for withdrawal (if applicable) 21
7.5 Replacement of individual subjects after withdrawal 21
7.6 Follow-up of subjects withdrawn from treatment 21
7.7 Premature termination of the study 21
8. SAFETY REPORTING 22
8.1 Section 10 WMO event 22
8.2 Adverse and serious adverse events 22
8.2.1 Suspected unexpected serious adverse reactions (SUSAR) 23
8.2.2 Annual safety report 23
8.3 Follow-up of adverse events 23
8.4 Data Safety Monitoring Board (DSMB) 23
9. STATISTICAL ANALYSIS 23

9.1 Descriptive statistics 24
9.2 Univariate analysis........ 23
9.3 Multivariate analysis........ 24
9.4 Interim analysis (if applicable)........ 24
10. ETHICAL CONSIDERATIONS 24
10.1 Regulation statement 24
10.2 Recruitment and consent 24
10.3 Objection by minors or incapacitated subjects (if applicable) 24
10.4 Benefits and risks assessment, group relatedness 24
10.5 Compensation for injury 25
10.6 Incentives (if applicable)........ 25
11. ADMINISTRATIVE ASPECTS AND PUBLICATION........ 26
11.1 Handling and storage of data and documents 26
11.2 Amendments 26
11.3 Annual progress report........ 26
11.4 End of study report 26
11.5 Public disclosure and publication policy 27
12. REFERENCES 27

LIST OF ABBREVIATIONS AND RELEVANT DEFINITIONS

**ABR**	ABR form, General Assessment and Registration form, is the application form that is required for submission to the accredited Ethics Committee (In Dutch, ABR = Algemene Beoordeling en Registratie)
**AE**	Adverse Event
**aHUS**	Atypical haemolytic uraemic syndrome
**AMD**	Age-related macular degeneration
**AP**	Alternative complement pathway
**AR**	Adverse Reaction
**CA**	Competent Authority
**CCM**	Central Committee on Research Involving Human Subjects; in Dutch: Centrale Commissie Mensgebonden Onderzoek
**CFH**	Complement factor H
**CNV**	Choroidal neovascularization
**CV**	Curriculum Vitae
**C3**	Complement C3
**C3d**	Complement C3d
**DSMB**	Data Safety Monitoring Board
**EU**	European Union
**EudraCT**	European drug regulatory affairs Clinical Trials
**GA**	Geographic atrophy
**GCP**	Good Clinical Practice
**IB**	Investigator's Brochure
**IC**	Informed Consent
**IMP**	Investigational Medicinal Product
**IMPD**	Investigational Medicinal Product Dossier
**METC**	Medical research ethics committee (MREC); in Dutch: medisch ethische toetsing commissie (METC)
**MG2**	Membranoproliferative glomerulonephritis type 2
**(S)AE**	(Serious) Adverse Event
**SPC**	Summary of Product Characteristics (in Dutch: officiële productinfomatie IB1-tekst)
**Sponsor**	The sponsor is the party that commissions the organisation or performance of the research, for example a pharmaceutical company, academic hospital, scientific

organisation or investigator. A party that provides funding for a study but does not commission it is not regarded as the sponsor, but referred to as a subsidising party.

SUSAR Suspected Unexpected Serious Adverse Reaction

Wbp Personal Data Protection Act (in Dutch: Wet Bescherming Persoonsgevens)

WMO Medical Research Involving Human Subjects Act (in Dutch: Wet Medisch-wetenschappelijk Onderzoek met Mensen

SUMMARY

Rationale: Zinc and antioxidants supplementation can delay the progression of age-related macular degeneration (AMD). The strongest genetic association for development of AMD has been found with the complement factor H (CFH) gene, which encodes a regulator of the complement cascade, a part of an innate immune system. Compared to controls, AMD patients have a higher level of complement-mediated inflammation as demonstrated by subretinal complement deposits (drusen). The AREDS study has demonstrated that zinc supplementation may prevent the progression of AMD and preserve visual function in 21 % of patients. In addition, it has been demonstrated that zinc has the ability to temper activation of the complement cascade by direct binding to active complement molecules.

Objective:

1. To determine if zinc supplementation in AMD patients has a direct measurable effect on the complement system explaining the mechanism through which this substance exerts its influence on AMD progression.
2. To determine whether this proposed effect of zinc is influenced by the genetic status, regarding the Y402H polymorphism in CFH, enabling us to identify subgroups of patients more susceptible to the beneficiary effect of zinc.

Study design: 51 AMD patients, 17 heterozygous and 17 homozygous carriers of the risk CFH genotype (CT and CC) as well as 17 homozygous nonrisk (TT) genotype will be enrolled. These groups will receive 50 mg oral zinc supplements during 3 months. Serum level of complement component C3 and activation fragments C3d will be analyzed prior, during and post treatment. In order to monitor the pharmacokinetic behaviour of zinc in the three patient groups, zinc levels will be determined in serum in time as the complement analyses. In case the zinc supplementation in AMD patients has a positive effect on complement parameters, we will like to obtain one venous blood extraction, two months after the ending of the zinc supplementation.

Study population: 51 AMD patients of 50 years of age or older with extensive small, intermediate, and large drusen, geographic atrophy and/or exudative AMD but without active disease as demonstrated by active neovascularisation, will be recruited for the study.

Intervention: All participants of the study will receive daily oral 50 mg zinc as zinc sulfate and 1 mg copper as cupric sulphate for 3 months. The reason for the presence of a small amount of copper is based on the fact that zinc and copper compete for the same membrane transport systems.

The ratio zinc to copper in the present preparation reflects the physiological situation. The same reasoning has also been followed in the Age-Related Eye Disease Study (AREDS) of the National Institutes of Health in the US.

Main study parameters/endpoints: The primary outcome is the serum level of activation fragment C3d and complement component C3, C3d/C3 ratio will be calculated. This ratio is the activity marker of alternative complement pathway.
The secondary outcome is the correlation between this supposed drop in serum level C3 en C3d and Y402H polymorphism status in CFH.

Nature and extent of the burden and risks associated with participation, benefit and group relatedness:
All participants of this 5 month study will be preselected from EUGENDA, a multi centre database for clinical and molecular analysis of age-related macular degeneration. At the first visit, after signing informed consent forms, each patient will undergo a routine ophthalmological examination, a determination of best corrected visual acuity using ETDRS charts. Furthermore, all patients will be examined by non invasive SD-OCT retina imaging. Zinc supplement will be given during the period of 3 months. In order to measure and monitor changes in serum complement levels at every visit (in total 4) venous blood will be collected. Serum zinc levels will be determined in the same samples. In order to detect symptoms that may indicate ongoing infection, at every visit the patient will undergo an interview (approximately 5 minutes). In case the zinc supplementation in AMD patients has a positive effect on complement parameters, two months after termination of zinc administration one more venous blood will de done and a 5 minute interview will be held. *(For more details see paragraph 7.3)*

Table 1: A list of all the assessments with 'x' indicating the time the assessment will be performed.
* In case of measurable positive effect of zinc on complement levels, the fifth visit will be schedule.

Visit number	1	2	3	4	5*
Study day	1	30	60	90	150*
PROCEDURE					
Information	x				
Informed consent	x				
Best-Corrected Visual Acuity (ETDRS)	x	x	x	x	x*
SD-OCT retina imaging	x				
Venous blood extraction	x	x	x	x	x*

5 min. interview	x	x	x	x	x*
Zinc supplementation	x	x	x	x	
End evaluation				x	

1. INTRODUCTION AND RATIONALE

Age-related macular degeneration (AMD) is the most common cause of irreversible severe visual loss in the developed world. The prevalence of AMD is 0.05% below the age of 50, rising to 11.8% above the age of 80.[1] Late AMD can be classified into two clinical subtypes. The wet or exudative type is characterized by choroidal neovascularization (CNV) under the retinal pigment epithelium (RPE) and/or neuroretina of the macula. These new vessels are weaker, resulting in a leakage, causing rapid devastation of vision.[2] The dry type with geographic atrophy (GA) is characterized by loss of retinal pigmented epithelium (RPE) and outer neurosensory retina cells. In both forms, drusen can be observed, most often in the posterior pole. These are pathological deposits of extracellular material formed between the RPE and Bruch membrane.[3] The disease mechanism of AMD is not fully understood. It is, however, a multifactorial disease in which multiple genetic and environmental factors are intertwined. Currently, the strongest genetic association for development of AMD that has been found is a polymorphism of the complement factor H (CFH) gene (Tyr402His), the inhibitor of the alternative complement pathway. Mutations and polymorphisms in this gene may negatively affect the functionality of factor H resulting in an abnormally high complement activity. The uncontrolled activation of the alternative pathway (AP) is probably a systemic immune reaction on expansive and cumulative oxidative stress in the aged outer retina.[4] It has been shown, for instance, that the blood complement levels in AMD patients are higher when compared to aged controls.[5] Innate immune system-mediated inflammation, specifically the activation of complement alternative pathway (AP) is possibly the earliest step in drusen formation and thus the development of AMD.[6,7]

The retina and the RPE are highly susceptible to oxidative stress because of elevated oxygen tension, high polyunsaturated lipid content, and high exposure to light. Zinc and copper function as cofactor in copper-zinc superoxide dismutase, a part of the primary antioxidant system used to regulate oxidative stress.[8,9] Furthermore, zinc induces the synthesis of metallothionins (MT), a group of proteins that have been shown to be scavenger of free radicals.[10] Post-mortem studies on human eyes have shown reduced levels of zinc and copper in RPE and the choroid complex in AMD eyes, as compared with age-matched controls.[11] In the Age-Related Eye Disease Study (AREDS), zinc supplementation had a beneficial effect on the progress of early AMD to advanced stages.[12] The combination of findings from different and independent studies suggest that zinc

homeostasis may play a role in AMD. However, the mechanism through which zinc influences the pathopfysiology of AMD is not yet understood. Different studies indicated that zinc, besides its antioxidant effects, also directly interacts with the complement cascades. It has been shown that zinc by itself has the ability to inhibit C3-convertasen (C3bBb) and the terminal stage of complement-mediated cytolysis by the membrane attack complex (MAC). When in vitro present simultaneously, CFH and zinc show a cumulative inhibition of alternative pathway.[13, 14]*(Figure 1)*
It has been demonstrated that food rich with zinc may protect against the increased risk of Tyr402His gene (CC genotype) for developing AMD early in the disease pathogenesis (L. Ho et al, ARVO 2009 abstract 1666; personal communication) One study indicated that an individual's response to AREDS supplement may be related to CFH genotype (TT, TC, CC). The main goal of this study is to test wither zinc supplementation has an effect on serum level of complement and provides insight into a beneficial effect of zinc in AMD patients. In this study a homozygous nonrisk genotype (TT) as compared to a homozygous risk genotype (CC) show a greater reduction in AMD progression.[15] The second goal of this study is to determine whether this proposed effect of zinc is influenced by the genetic status, regarding the Y402H polymorphism in CFH.

Figure 1: interaction between alternative complement pathway and zinc

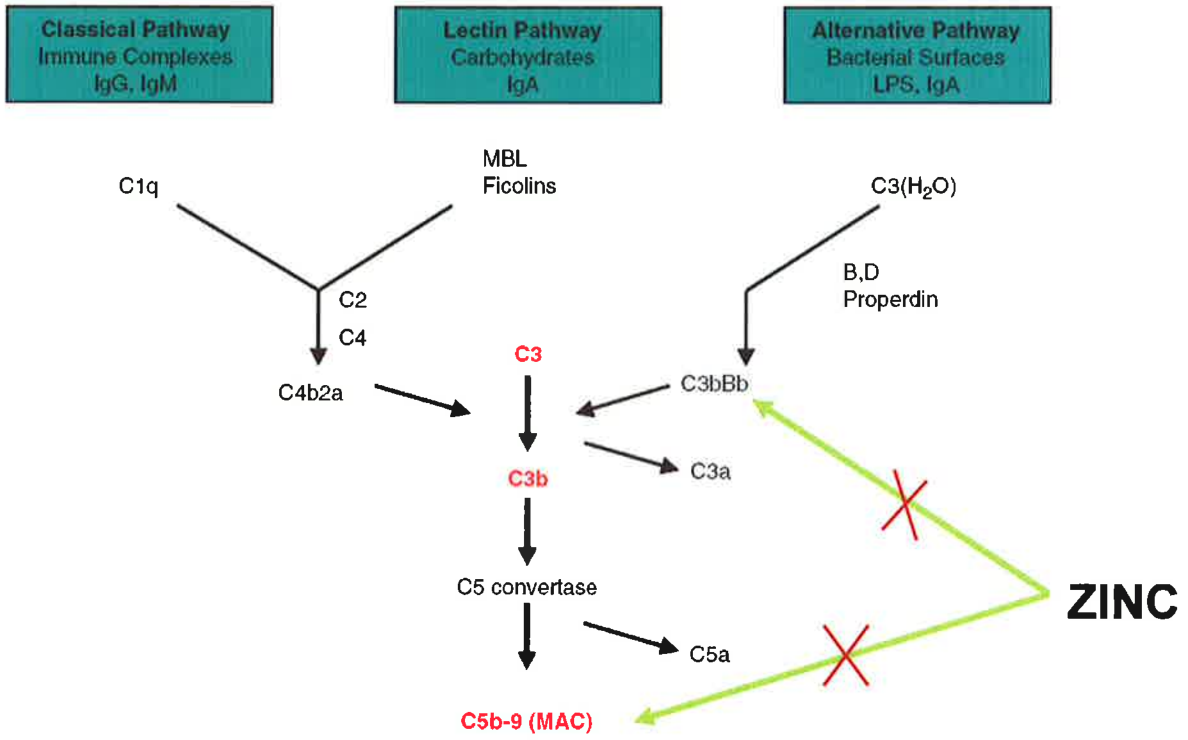

2. OBJECTIVES

Primary Objective: To determine if zinc supplementation in AMD patients has a direct measurable effect on the complement system explaining the mechanism through which this substance exerts its influence on AMD progression.

Secondary Objective: To determine whether this proposed effect of zinc is influenced by the genetic status, regarding the Y402H polymorphism in CFH, enabling us to identify subgroups of patients more susceptible to the beneficiary effect of zinc.

3. STUDY DESIGN

This is a 5 month open label study on 51 AMD patients, preselected from a EUGENDA, a multi centre database for clinical and molecular analysis of age-related macular degeneration. Participants will be treated with orally 50 mg zinc and 1 mg copper daily during 3 months. Serum complement and zinc levels will be measured from extracted venous blood prior to treatment and monthly during the treatment. Primary endpoint will be a change in serum C3d and C3 level (C3d/C3 ratio) after 3 months en of treatment as compared to the levels prior treatment. The secondary endpoint outcome is the correlation between this supposed drop in serum level C3d and Y402H polymorphism status in CFH. In case the zinc supplementation in AMD patients decreases the complement levels, two months after the ending of the zinc one more venous blood extraction (in total 5) and 5 minute interview will be obtained.

4. STUDY POPULATION

4.1 Population

Fifty-one AMD patients, 17 heterozygous, and 17 homozygous carriers of the CFH Y402H gene variant and 17 non carriers. *(For more details please see paragraph 4.4)*

4.2 Inclusion criteria

- Men and women ≥ 50 years of age.
- AMD patients previously included in the EUGENDA database.
- Previously genotyped for Y402H (rs1061170) gene variation (from EUGENDA database).
- Patients with extensive small drusen, intermediate drusen, large drusen, advanced neovascular AMD without neovascular activity in one or both eyes or geographic atropy in one or both eyes.
- Informed consent.

4.3 Exclusion criteria

- Active leakage from CNV due to AMD.
- Ongoing anti/VEGF treatment.
- Ongoing infection.
- Subretinal hemorrhages.
- History of any vitreous hemorrage within 12 weeks.
- Other ocular disorders that may confound the interpretation of the study results.
- Systemic or local steroid treatment within the last three months.
- Use of any antibiotica.
- Prolonged use of diuretics.
- Supplemental use of iron (38-65 mg/day of elemental iron).
- Use of zink and vitamin supplements one month prior to the study.
- Systhemic diseases that may influence complement levels (atypical haemolytic uraemic syndrome (aHUS), membranoproliferative glomerulonephritis type 2 (MG2)).

4.4 Sample size calculation

In our database, the mean level of complement factor C3d is 15.7 µg/ml for AMD patients and 11.9 µg/ml for controls. There are no current data to help us predict the decrease, if any, in factor C3d. We assume that a decrease of 30% in serum complement level in AMD patients should be possible. This would mean that the C3d level in AMD patients would decrease with 1.2 µg/ml or an 8% decrease compared to the baseline level. A simulation of that scenario resulted in an estimated standard deviation of differences of 1.4 µg/ml. A paired t-test with a two-sided significance level of 5% in a sample size of 14 patients would have 80% power to detect a difference in means of 1.2 µg/ml, assuming a standard deviation of differences of 1.4 µg/ml. Taking into consideration a 20% drop-out rate or noncompliance we need to invite 17 AMD patients to maintain the same power in the study. The level of C3d, a marker of chronic activation of complement cascade, shows a possible dose-response relationship with CFH genotype. *Figure* 2 (D. Smailhodzic et al., ARVO 2010 abstract 6201, *our unpublished data)* To determine whether the genetic status regarding CFH interacts with the proposed effect of zinc, we need to invite 17 noncarriers, 17 heterozygous carriers, and 17 homozygous carriers of CFH Y402H gene variant.

Figure 2: C3d levels clustered by CFH genotype (green=controls, red=AMD patients)

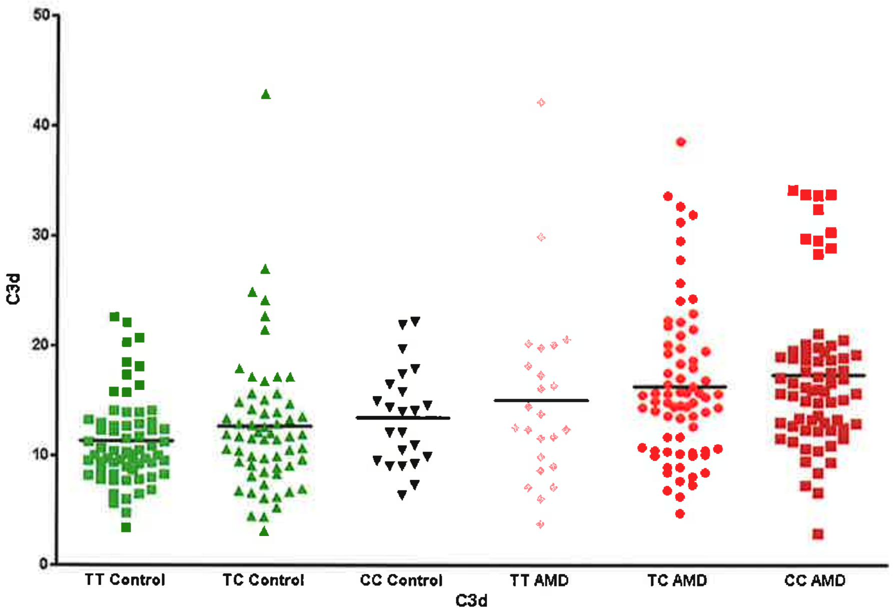

5. TREATMENT OF SUBJECTS

5.1 Investigational product/treatment

All study participants will be treated with 50 mg zinc as zinc sulphate orally. Zinc sulphate is commonly used to treat or prevent low levels of zinc. The major consequence of long-term consumption of excessive zinc is copper deficiency. In order to prevent copper deficiency, 1 mg copper as cupric sulphate will be added to the treatment.

5.2 Use of co-intervention

Advice is to take in the zinc sulphate 1 hour before or 2 hours after meals but it may be taken with food if it upsets a stomach. Use of additional oral antioxidants and vitamin supplementation may influence the study outcomes. These substances are not allowed, starting one month prior to treatment. Concomitant administration of zinc supplements and certain antibiotics, specifically tetracyclines and quinolones, may decrease absorption of the antibiotic and potentially reduce its efficacy. Furthermore, ongoing infection may cause changes in the complement levels. Therefore all AMD patient using antibiotics will be excluded from our study. Prolonged use of diuretics may increase urinary zinc excretion, resulting in increased loss of zinc: these patients will be excluded. Supplemental (38-65 mg/day) of elemental iron, but not dietary levels (8 mg/day) of iron may decrease zinc absorption.[16] AMD patient using supplemental iron will be also excluded from our study.

5.3 Escape medication

Not applicable.

6. INVESTIGATIONAL MEDICINAL PRODUCT

6.1 Name and description of investigational medicinal product(s)

Zinc sulfate and copper sulfate are a mineral, commonly used as a food supplement and freely available on the market without medical prescription. This study will not make use of a medicinal product. The AREDS study used 80 mg zinc daily and did not observe serious side effects. In this study we use a doses lower than the doses used in the clinical studies.
The zinc preparation to used in the present study fully complies to the requirements of the European Pharmacopeia (EP) and the United States Pharmacopeia (USP). Details can be found on the attached monographs taken from both the EP and the USP. This means that lack of contaminating and/or adverse substances can be guaranteed. Analytical purity is almost 100%. (Please seem K4, European Pharmacopoeia 5.0, 'Zinc sulphate heptahydrate') Eventual minuscule impurities are known from the analytical analysis dossier and only if shown to be irrelevant and harmless, the Pharmacopeia quality certification is given. In a number of countries (e.g. Germany) this type of preparation is available as registered over-the-counter pharmaceutical drug. In most countries, however, zinc is available as nutritional supplement, mostly in combination with copper and other minerals and/or vitamins. The form in which zinc is going to be used in the present study also features on the most recent list of the European Food Safety Authority (EFSA), the EU-Institution that controls and guarantees the safety of food components.
What has been stated here regarding the zinc preparation is also fully applicable to the copper compound (cupric sulphate) that is incorporated in the zinc supplement in a very small amount.

6.2 Summary of findings from non-clinical studies

Not applicable.

6.3 Summary of findings from clinical studies

Pease see chapter 6.4 and 6.5.

6.4 Summary of known and potential risks and benefits

Risks of zinc supplementation:

Mild gastrointestinal distress has been reported at doses of 50 to 150 mg/day of supplemental zinc and symptoms cease immediately after cessation of intake.[17] Increased hospital admission due to genitourinary problems, such as unspecified urinary tract infections and prostatic hyperplasia in men and stress incontinence in women.[18] Debate is going on regarding the recommended daily allowance (RDA) of zinc. The average intake in the population in the EU is around 10 mg per day. The amount is used as RDA in the most countries is 15 mg per day. This implies that, generally spoken, one third of the population has a sub-adequate intake of zinc. This is especially the case in the elderly, since in this group there is an additional complicating factor: decreased resorption. (Biesalski et al., 2004. "Ernährungsmedizin", Thieme Verlag, Stuttgart). There are no reports on adverse reactions in the context of extra intake of 50 mg zinc. This is confirmed by the data from the ARED study, where a daily dose of 80 mg was used. The major consequence of long-term consumption of excessive zinc is copper deficiency. One of the most common clinical signs of copper deficiency is an anemia that is unresponsive to iron therapy but corrected by copper supplementation. Copper deficiency may also result in abnormally low numbers of white blood cells known as neutrophils (neutropenia).[19] In order to prevent copper deficiency, 1 mg copper will be added to the treatment.

Benefits of zinc supplementation:

Zinc supplementation is being used in the prevention of diarrhea and pneumonia and in achieving optimal growth.[20, 21] Zinc supplementation is associated with decreased morbidity and mortality in children and elderly.[12, 22-24] There was significant reduction in AMD progression in AMD high-risk individuals, estimates of derivated from Odds suggest risk reduction for those patients taking zinc alone of 21%.[12]

Risks of copper supplementation:

Symptoms of acute copper toxicity include abdominal pain, nausea, vomiting, and diarrhea, which help prevent additional ingestion and absorption of copper. More serious signs of acute copper toxicity include severe liver damage, kidney failure, coma, and death. In generally healthy individuals, doses of up to 10,000 mcg (10 mg) daily have not resulted in liver damage. The U.S. Food and Nutrition Board (FNB) set the tolerable upper level of intake (UL) for copper at 10 mg/day from food and supplements. *(Please see K4, Scientific commission on food 2004, Copper Safety)*We believe that is a highly unexpected that 1 mg copper, used in this study, will cause a copper toxicity.

6.5 Description and justification of route of administration and dosage

Zinc homeostasis is highly via the gastrointestinal tract Zinc absorption is concentration dependent and occurs throughout the small intestine. The jejunum has the highest rate of absorption of zinc.[25, 26] The amount of zinc in a meal will, in itself, affect zinc absorption [27, 28] With increasing amounts of zinc in a meal, fractional zinc absorption (%) will decrease. Advice is to take zinc sulfate 1 hour before or 2 hours after the meals. But if this mineral upsets a stomach it may be taken with food.

The Food and Nutrition Board (FNB) indentified NOAEL (no observable adverse effect level) for zinc of approximately 50 mg per day and LOAEL (lowest observed adverse effect level) of approximately 60 mg per day. *(Please see K4, appendix 2; Scientific commission on food 2003)* Estimation of total zinc intakes of the study participants is 60 mg/day, 50 mg supplemental and 10 mg dietary (FNB estimation). Clinical trial AREDS, which demonstrated a delay in AMD progression, used 80 mg zinc and 2 mg of copper.[12] Study of the absorption of zinc sulfate in healthy adults concluded that zinc doses of more than 20 mg result in relatively small and progressively diminishing increase in aqueous zinc.[29] Taking into consideration the findings from those two studies, in order to minimize a side effects and maximize a zinc absorption, we chose a zinc supplement dose of 50 mg/day and of 1 mg copper. *Figure 3*

Figure 3: Individual measurements of absorbed zinc (AZ) versus ingested zinc (IZ)

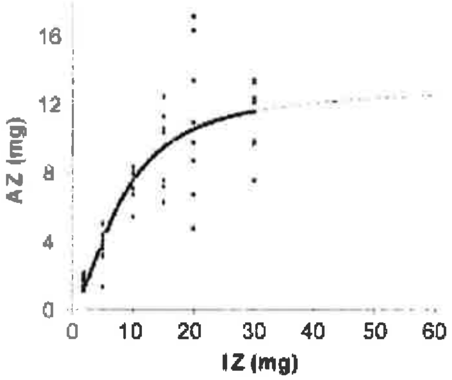

6.6 Dosages, dosage modifications and method of administration

For this study we will use zinc and copper supplement capsules originating from company 'Sanmed'. This company has a long standing experience in the development of nutritional supplements incorporating ingredients with Pharmacopieia monograph quality. The supplement for patients with AMD, based on the formula of the ARED study ("Macula Support"), is a product from this company.

6.7 Preparation and labelling of Investigational Medicinal Product

The investigational product is not a medicinal product, but a nutritional supplement. This means that it is regulated by an EU-Directive (copy attached) and not by pharmaceutical law. Nutritional supplements are no drugs, but "food-stuffs". In The Netherlands they under the jurisdiction of the "Voedsel en Warenautoriteit". Generally, nutritional supplements are freely available. No explicit claims for indications are allowed to be made, unless EFSA permits the use of such claims, which must be based on scientific evidence and clinical studies. (*See F4, Richtlijn 2002/46/RG Van het Europese parlament en de Raad)*

The capsules with zinc and copper to be used in the present study are specially prepared for this purpose. The manufacturing, however, uses standard pharmacological methods and GMP pharmaceutical technology.

The capsule material is of non-animal origin, thus obviating issues associated with vegetarians, kosher and hallal. The shell of the capsules is made of a processed form of cellulose (hydroxypropyl methylcellulose, HPMC). This nmamaterila is widely known throughout he pharmaceutical industry, where it is used for coating of tablets and for easily digestible capsules .

6.8 Drug accountability

The nutritional supplement to be used in the present study is specially made for this purpose. However, it is manufactured under the same GMP conditions as is the case with the other product of Sanmed. The product mentioned above ("Macula Support") contains comparable amounts of zinc and copper, in addition to anti-oxidative vitamins. Since its introduction 5 year ago more than 1 million capsules have been sold (and used). Accoridng o theQuality System of Sanmed, no adverse reactions have occurred, except for light nausea complaints in 15 to 20 patients.

In case of reclamations, the Tracebility procedure of the Sanmed Quality System will follow-up on the complaint and the steps needed for the individual measures are taken and reported to the "Voedsel en Warenautoriteit". Part of this system is also a Liability Insurance.

7. METHODS

7.1 Study parameters/endpoints

7.1.1 Main study parameter/endpoint

The primary outcome is the serum level of activation fragment C3d and complement component C3, C3d/C3 ratio will be calculated. This ratio is the activity marker of

alternative complement pathway.

7.1.2 Secondary study parameters/endpoints (if applicable)

The secondary outcome is the correlation between this supposed drop in serum level C3d and Y402H polymorphism status in CFH.

7.1.3 Other study parameters (if applicable)

The levels of zinc in serum will be used to assess the uptake of the zinc.

7.2 Randomisation, blinding and treatment allocation

In this study randomisation procedure will not be used.

7.3 Study procedures

Table one summarizes procedures that will be conducted for each patient during the study.

Table 1: A list of all the assessments with 'x' indicating the time the assessment will be performed. * In case of measurable positive effect of zinc on complement levels, the fifth visit will be schedule.

Visit number	1	2	3	4	5*
Study day	1	30	60	90	150*
PROCEDURE					
Information	x				
Informed consent	x				
Best-Corrected Visual Acuity (ETDRS)	x	x	x	x	x*
SD-OCT retina imaging	x				
Venous blood extraction	x	x	x	x	x*
5 min. interview	x	x	x	x	x*
Zinc supplementation	x	x	x	x	
End evaluation				x	

Best corrected visual acuity

Visual acuity (VA) will be assessed of both eyes at the first and last study visit. VA measurement will be taken using ETDRS visual acuity testing chart at an initial testing distance of 4 meters.

SD-OCT Imaging

All patients will be examined by non invasive SD-OCT retinal imaging. This imaging technique is well suited to observe a neovascular activity. This examination is quick (3 minutes) and there is no discomfort to the patient besides the pupillary dilation prior to the scan (with1.0% tropicamide and 2.5% phenylephrine). Note: not all patients may need dilation, only with small pupil size. OCT imaging will be carried out with a Spectralis HRA+OCT, Heidelberg Engineering, Heidelberg, Germany.

Blood sample collection

For each patient the following venous blood samples will be collected for molecular analysis:

- 1 x 10 ml coagulation tube for serum collection
- 1 x 5 ml heparin blood

List of molecular analysis:

- complement activation fragment C3d
- complement component C3
- zinc serum level
- C- reactive protein (CRP)

Justification of the 2 blood tubes:

The test of the alternative complement pathway needs to be done in the serum. Zink levels can be measured from extracted serum and C- reactive protein (CRP) from heparin blood. Therefore two tubes of venous blood are needed.

Concomitant infection screening:

Ongoing infection may cause change in the status of the complement level. To check for inflammation in the body standard serum test (CRP) will be perform. The symptoms that may indicate a ongoing infection will be obtained additionally during a quick (5 min.) interview. *(For interview please see F1)*

Justification of monthly venous blood collection:

There is little known about the dynamics of zinc and the interactions of zinc supplementation on complement cascade. Furthermore, complement levels can fluctuate over the time, especially in periods of infection. For this reason, we believe that a minimum of three measurements during the three month supplementation period can be justified.

Justification of one venous blood collection two months post supplementation:
In case zinc supplementation in AMD patients has a positive effect on the complement system, it will be interesting to test whether complement status in the absence of zinc returns to the previously higher activation level. Therefore we would like to perform one venous blood extraction, approximately two months after the ending of the zinc supplementation.

7.4 Withdrawal of individual subjects

Subjects can leave the study at any time for any reason if they wish to do so without any consequences. The investigator can decide to withdraw a subject from the study for urgent medical reasons.

7.4.1 Specific criteria for withdrawal (if applicable)

Not applicable.

7.5 Replacement of individual subjects after withdrawal

In sample size calculations we took into consideration that approximately 20% of the study individuals will not use the zinc supplements according to prescription or will drop-out from the study. The reason for this consideration is the high age of the AMD population with a mean age of 76 years, often with impaired visual site and poor mobility.

7.6 Follow-up of subjects withdrawn from treatment

Not applicable.

7.7 Premature termination of the study

In this study we use a food supplement that is freely available over the counter and in a doses lower than the doses used in the clinical studies. The AREDS study used 80 mg zinc daily and did not observe serious side effects. It is a highly unexpected that 50 mg zinc and 1 mg copper, used in this study, will cause a serious side effects. Making the safety of our patients our priority all study individual will be instructed to contact us in any case of unsuspected side affect. All adverse events reported spontaneously by the subject or observed by the investigator or his staff will be recorded. All SAEs will be reported to the central METC. In case of occurrence of (serious) adverse events and/or a high number of

patients who end zinc intake prematurely, the principal investigator will be responsible for the premature termination of the study.

8. SAFETY REPORTING

8.1 Section 10 WMO event

In accordance to section 10, subsection 1, of the WMO, the investigator will inform the subjects and the reviewing accredited METC if anything occurs, on the basis of which it appears that the disadvantages of participation may be significantly greater than was foreseen in the research proposal. The study will be suspended pending further review by the accredited METC, except insofar as suspension would jeopardise the subjects' health. The investigator will take care that all subjects are kept informed.

8.2 Adverse and serious adverse events

Adverse events are defined as any undesirable experience occurring to a subject during the study, whether or not considered related to [the investigational product / the experimental treatment]. All adverse events reported spontaneously by the subject or observed by the investigator or his staff will be recorded.

A serious adverse event is any untoward medical occurrence or effect that at any dose:

- results in death;
- is life threatening (at the time of the event);
- requires hospitalisation or prolongation of existing inpatients' hospitalisation;
- results in persistent or significant disability or incapacity;
- is a congenital anomaly or birth defect;
- is a new event of the trial likely to affect the safety of the subjects, such as an unexpected outcome of an adverse reaction, lack of efficacy of an IMP used for the treatment of a life threatening disease, major safety finding from a newly completed animal study, etc.

All SAEs will be reported through the web portal *ToetsingOnline* to the accredited METC that approved the protocol.

SAEs that result in death or are life threatening should be reported expedited. The expedited reporting will occur not later than 7 days after the responsible investigator has first

knowledge of the adverse reaction. This is for a preliminary report with another 8 days for completion of the report.

8.2.1 Suspected unexpected serious adverse reactions (SUSAR)

Not applicable.

8.2.2 Annual safety report

Not applicable, this is 5 months study.

8.3 Follow-up of adverse events

All adverse events will be followed until they have abated, or until a stable situation has been reached. Depending on the event, follow up may require additional tests or medical procedures as indicated, and/or referral to the general physician or a medical specialist.

8.4 Data Safety Monitoring Board (DSMB)

For this non-commercial research the DSMB will not be established. Ongoing safety surveillance and interim analyses on the safety data, will be performed by the investigator and medical staff involved in this study. The investigator will report the occurrences of serious adverse events.

9. STATISTICAL ANALYSIS

9.1 Descriptive statistics

All data will be quantitatively presented. Patient characteristics and treatment outcomes will be presented in percentages or means with standard deviations (completed with percentiles where appropriate). The primary outcome measure will be the difference in serum complement levels between baseline and 3 months.

9.2 Univariate analysis

The purpose of this study is to study the difference between serum complement levels prior and after zinc supplementation at 3 months. A paired design is very powerful in detecting differences: the paired t-teat will be used to determine the difference between the baseline measurement and the measurement at 3 months. ANOVA will be used to determine a difference in components of the serum complement level between the groups.

9.3 Multivariate analysis

Multivariate analysis with logistic regression analysis will be applied to study independence and interaction of explanatory variables.

9.4 Interim analysis

Not applicable.

10. ETHICAL CONSIDERATIONS

10.1 Regulation statement

This study will be conducted according to the principles of the Decleration of Helsinki (version 4) and in accordance with the Medical research Involving Human Subjects Act (WMO) and other guidelines, regulation and Acts. Data management, monitoring and reporting of the study will be performed in accordance with the ICH GCP guidelines.

10.2 Recruitment and consent

All participants of this 5 month study will be preselected from a EUGENDA, a multi centre database for clinical and molecular analysis of age-related macular degeneration. All subjects included in this database signed a written informed consent for data collection and future studies data use. Suitable patients will be contact and asked if they wish to participate in this study. Information material will be sent to the patient and approximately one week later an appointment with coordinating investigator, for extra information and a inform consent, will be planed. The patients may only be included in this study after a written informed consent has been obtained. The patient should indicate consent by personally signing and dating the written informed consent document. *(For patients information's and informed consent please see E1 and E2)* After signing a informed consent standard ophthalmological examination will be carried out and a series of three extra visit to ophthalmology clinic at UMC St. Radboud will be planed.

10.3 Objection by minors or incapacitated subjects

Minors and legally incompetent adults are excluded from the study.

10.4 Benefits and risks assessment, group relatedness

It is known that supplementation with zinc and antioxidants delay the progression of

AMD. In vitro studies have demonstrated that zinc has the ability to temper activation of the complement cascade by direct binding to active complement molecules. The clinical relevance of this study is to investigate wither treatment with zinc has the potency to lower complement mediated inflammation in AMD patients. This might provide the mechanism through which zinc exerts its protective effects. In addition the underlying pharmacogenetics are studied by linking the CFH polymorphism profile to the effect of zinc on complement levels. In this way, genetically defined patients groups can be targeted for zinc supplementation thus avoiding over- and undertreatment. In this study we use a food supplement freely available on the market and in the dosage lower than previously clinically tested. AREDS study did not observe any serious side effects. We studied available literature with great accuracy and we concluded that it is a highly unexpected that the 50 mg zinc and 1 mg copper, used in this study, will cause SAE.

10.5 Compensation for injury

The sponsor/investigator has a liability insurance which is in accordance with article 7, subsection 6 of the WMO.

UMC St Radboud medical research has an insurance which is in accordance with the legal requirements in the Netherlands (Article 7 WMO and the Measure regarding Compulsory Insurance for Clinical Research in Humans of 23th June 2003). This insurance provides cover for damage to research subjects through injury or death caused by the study.

1. € 450.000,-- (i.e. four hundred and fifty thousand Euro) for death or injury for each subject who participates in the Research;
2. € 3.500.000,-- (i.e. three million five hundred thousand Euro) for death or injury for all subjects who participate in the Research;
3. € 5.000.000,-- (i.e. five million Euro) for the total damage incurred by the organisation for all damage disclosed by scientific research for the Sponsor as 'verrichter' in the meaning of said Act in each year of insurance coverage.

The insurance applies to the damage that becomes apparent during the study or within 4 years after the end of the study.

10.6 Incentives

Enrolled patients will receive lunch and travelling expenses for the visits at our clinic.

11. ADMINISTRATIVE ASPECTS AND PUBLICATION

11.1 Handling and storage of data and documents

The investigator will set up a trial master File at the beginning of the study. The list of essential documents will be in accordance with the GCP-guidelines. The essential documents that make up the file will be stored in a secure but accessible manner. All essential documents will be legible and accurate. Patients data will be handled confidentially and in case of blood measurements anonymously. The identification code list will be made and kept by the investigator. The handling of all personal data will be in accordance with the Dutch Personal Data Protection Act. After the end of the study all essential documents pertaining to the conduct of the study, (e.g., screening forms, patients files, originals of test result reports, correspondence, records of informed consent, etc) will be archived by the investigator for a period of 15 years in accordance with the standard operating procedure of the UMC St. Radboud.

11.2 Amendments

Amendments are changes made to the research after a favourable opinion by the accredited METC has been given. All amendments will be notified to the METC that gave a favourable opinion.

11.3 Annual progress report

The sponsor/investigator will submit a summary of the progress of the trial to the accredited METC once a year. Information will be provided on the date of inclusion of the first subject, numbers of subjects included and numbers of subjects that have completed the trial, serious adverse events/ serious adverse reactions, other problems, and amendments.

11.4 End of study report

The investigator will notify the accredited METC of the end of the study within a period of 8 weeks. The end of the study is defined as the last patient's last visit.

In case the study is ended prematurely, the investigator will notify the accredited METC, including the reasons for the premature termination.

Within one year after the end of the study, the investigator/sponsor will submit a final

study report with the results of the study, including any publications/abstracts of the study, to the accredited METC.

11.5 Public disclosure and publication policy

This study is a non-commercial research, data of scientific value will be published.

12. REFERENCES: reference list

1. Friedman DS, O'Colmain BJ, Munoz B et al. Prevalence of age-related macular degeneration in the United States
   6. Arch Ophthalmol 2004; 122(4):564-572.

2. Gass JD, Agarwal A, Lavina AM, Tawansy KA. Focal inner retinal hemorrhages in patients with drusen: an early sign of occult choroidal neovascularization and chorioretinal anastomosis
   1. Retina 2003; 23(6):741-751.

3. de Jong PT. Age-related macular degeneration. N Engl J Med 2006; 355(14):1474-1485.

4. Hollyfield JG, Bonilha VL, Rayborn ME et al. Oxidative damage-induced inflammation initiates age-related macular degeneration. Nat Med 2008; 14(2):194-198.

5. Scholl HP, Charbel IP, Walier M et al. Systemic complement activation in age-related macular degeneration
   22. PLoS One 2008; 3(7):e2593.

6. Anderson DH, Mullins RF, Hageman GS, Johnson LV. A role for local inflammation in the formation of drusen in the aging eye
   33. Am J Ophthalmol 2002; 134(3):411-431.

7. Johnson LV, Leitner WP, Staples MK, Anderson DH. Complement activation and inflammatory processes in Drusen formation and age related macular degeneration. Exp Eye Res 2001; 73(6):887-896.

8. Bell SG, Vallee BL. The metallothionein/thionein system: an oxidoreductive metabolic zinc link
   5. Chembiochem 2009; 10(1):55-62.

9. Tate DJ, Newsome DA. A novel zinc compound (zinc monocysteine) enhances the antioxidant capacity of human retinal pigment epithelial cells
   3. Curr Eye Res 2006; 31(7-8):675-683.

10. Sato M, Bremner I. Oxygen free radicals and metallothionein
    11. Free Radic Biol Med 1993; 14(3):325-337.

11. Erie JC, Good JA, Butz JA, Pulido JS. Reduced zinc and copper in the retinal pigment epithelium and choroid in age-related macular degeneration. Am J Ophthalmol 2009; 147(2):276-282.

12. A randomized, placebo-controlled, clinical trial of high-dose supplementation with vitamins C and E, beta carotene, and zinc for age-related macular degeneration and vision loss: AREDS report no. 8. Arch Ophthalmol 2001; 119(10):1417-1436.

13. Blom AM, Kask L, Ramesh B, Hillarp A. Effects of zinc on factor I cofactor activity of C4b-binding protein and factor H
3. Arch Biochem Biophys 2003; 418(2):108-118.

14. Yamamoto K, Takahashi M. Inhibition of the terminal stage of complement-mediated lysis (reactive lysis) by zinc and copper ions. Int Arch Allergy Appl Immunol 1975; 48(5):653-663.

15. Klein ML, Francis PJ, Rosner B et al. CFH and LOC387715/ARMS2 genotypes and treatment with antioxidants and zinc for age-related macular degeneration
22. Ophthalmology 2008; 115(6):1019-1025.

16. Sandstrom B. Micronutrient interactions: effects on absorption and bioavailability
26. Br J Nutr 2001; 85 Suppl 2:S181-S185.

17. Sriram K, Lonchyna VA. Micronutrient supplementation in adult nutrition therapy: practical considerations
4. JPEN J Parenter Enteral Nutr 2009; 33(5):548-562.

18. Johnson AR, Munoz A, Gottlieb JL, Jarrard DF. High dose zinc increases hospital admissions due to genitourinary complications. J Urol 2007; 177(2):639-643.

19. Fujita M, Itakura T, Takagi Y, Okada A. Copper deficiency during total parenteral nutrition: clinical analysis of three cases
1. JPEN J Parenter Enteral Nutr 1989; 13(4):421-425.

20. Bhutta ZA, Black RE, Brown KH et al. Prevention of diarrhea and pneumonia by zinc supplementation in children in developing countries: pooled analysis of randomized controlled trials. Zinc Investigators' Collaborative Group. J Pediatr 1999; 135(6):689-697.

21. Brown KH, Peerson JM, Allen LH. Effect of zinc supplementation on children's growth: a meta-analysis of intervention trials. Bibl Nutr Dieta 1998;(54):76-83.

22. Baqui AH, Black RE, El AS et al. Effect of zinc supplementation started during diarrhoea on morbidity and mortality in Bangladeshi children: community randomised trial. BMJ 2002; 325(7372):1059.

23. Black RE. Therapeutic and preventive effects of zinc on serious childhood infectious diseases in developing countries
168. Am J Clin Nutr 1998; 68(2 Suppl):476S-479S.

24. Meydani SN, Hamer DH. Zinc supplementation in elderly nursing home residents. Clin Infect Dis 2009; 49(3):479.

25. Lee HH, Prasad AS, Brewer GJ, Owyang C. Zinc absorption in human small intestine. Am J Physiol 1989; 256(1 Pt 1):G87-G91.

26. Tuerk MJ, Fazel N. Zinc deficiency
1. Curr Opin Gastroenterol 2009; 25(2):136-143.

27. Sandstrom B. Bioavailability of zinc. Eur J Clin Nutr 1997; 51 Suppl 1:S17-S19.

28. Sandstrom B, Arvidsson B, Cederblad A, Bjorn-Rasmussen E. Zinc absorption from composite meals. I. The significance of whest extraction rate, zinc, calcium, and protein content in meals based on bread. Am J Clin Nutr 1980; 33(4):739-745.

29. Tran CD, Miller LV, Krebs NF, Lei S, Hambidge KM. Zinc absorption as a function of the dose of zinc sulfate in aqueous solution. Am J Clin Nutr 2004; 80(6):1570-1573.
